# Cytotoxic activity of marine derived bioactive compounds from red sea sponges supported by LC-MS/MS profiling and molecular docking

**DOI:** 10.1038/s41598-026-39782-z

**Published:** 2026-03-12

**Authors:** Noha E. Ibrahim, Amal M. El-Feky, Mohamed Aboelmagd, Nadia A. Mohammed, Rehab A. Mohamed, Ahmed A. El-Rashedy, Hanaa M. Rady

**Affiliations:** 1 Microbial Biotechnology Department, Biotechnology Research Institute, National Research Centre, 33 El Bohouth St. (Former El Tahrir St.), P.O. 12622, Dokki, Giza, Egypt; 2 Pharmacognosy Department, National Research Centre, 33 El Bohouth St. (Former El Tahrir St.), P.O. 12622, Dokki, Giza, Egypt; 3 Medical Biochemistry Department, National Research Centre, 33 El Bohouth St. (Former El Tahrir St.), P.O. 12622, Dokki, Giza, Egypt; 4 Natural and Microbial Products Department, National Research Center, 33 El Bohouth St. (Former El Tahrir St.), P.O. 12622, Dokki, Giza, Egypt; 5 Department Organic and Medicinal Chemistry, Faculty of Pharmacy, University of Sadat City, Menoufia, 32897 Egypt; 6 Chemistry of Natural Compounds Department, National Research Centre, 33 El Bohouth St. (Former El Tahrir St.), P.O. 12622, Dokki, Giza, Egypt

**Keywords:** Marine sponges, *Stylissa carteri*, Cytotoxicity, Pyrrole-imidazole alkaloids, Molecular docking, Biochemistry, Biotechnology, Cancer, Chemical biology, Chemistry, Computational biology and bioinformatics, Drug discovery

## Abstract

Marine sponges are prolific sources of structurally diverse secondary metabolites with notable pharmacological potential. This study aimed to explore and compare the chemical composition and cytotoxic activity against liver cancer cells of three Red Sea sponge species—*Stylissa carteri*, *Hemimycale arabica*, and *Negombata magnifica*—collected from distinct ecological sites. Sequential solvent extraction was conducted using n-hexane, ethyl acetate, and n-butanol, followed by phytochemical screening, and LC–MS/MS-based metabolite profiling. Cytotoxicity was evaluated through MTT assay, colony formation, and wound healing (scratch) assay against HepG2 liver cancer cells. Molecular docking was carried out using the Chk2 kinase receptor to assess binding affinities and interaction modes of the identified metabolites. The total MeOH:DCM extract of *S. carteri* (El Gouna) showed potent cytotoxicity, with an IC_50_ of 37 µg/mL, 80% growth inhibition in the MTT assay, and significant suppression of colony formation (20.3 ± 2.1% survival) and cell migration (35.4 ± 2.1% wound closure). LC–MS/MS analysis tentatively identified pyrrole–imidazole alkaloids, including hymenialdisine, oroidin, and spongiacidin D. Docking revealed strong binding affinities of hymenialdisine (− 8.35 kcal/mol) and spongiacidin D (− 7.30 kcal/mol) with key Chk2 active-site residues. MD simulations indicated that the hymenialdisine–Chk2 complex maintained stability throughout 100 ns, with reduced RMSD (2.54 ± 0.48 Å), RMSF (1.46 ± 0.14 Å), Rg (20.70 ± 0.17 Å), and SASA (13,916 Å^2^) compared to the Apo protein, confirming a rigid and stable ligand–protein interaction. MM/GBSA calculations revealed a favorable total binding free energy (ΔG_bind =  − 33.17 ± 0.17 kcal/mol), dominated by van der Waals and electrostatic contributions. ADMET predictions indicated compliance with Lipinski and Veber rules, suggesting good oral bioavailability. *S. carteri* from El Gouna is enriched in bioactive brominated alkaloids that exhibit potent cytotoxic activity against HepG2 cells. Integration of cytotoxic assays, LC–MS/MS profiling, docking, and MD simulations underscores the influence of species and environmental conditions on sponge-derived bioactivity. These findings provide a strong rationale for advancing *S. carteri* metabolites, especially hymenialdisine and spongiacidin D as potential cytotoxic agents.

**Supplementary Information:**

The online version contains supplementary material available at 10.1038/s41598-026-39782-z.

## Introduction

Marine sponges (phylum *Porifera*) are renowned for their remarkable ability to produce structurally diverse secondary metabolites that play critical roles in chemical defense, ecological adaptation, and interspecies interactions^1^. These metabolites, including alkaloids, terpenoids, polyketides, peptides, and sterols, demonstrate a wide spectrum of pharmacological activities, such as anticancer, antimicrobial, antiviral, and anti-inflammatory effects^2–4^. The remarkable chemical diversity observed in marine sponges is largely attributed to their evolutionary adaptation to complex and competitive marine environments, as well as to their symbiotic relationships with bacteria, fungi, and cyanobacteria, which often serve as biosynthetic partners^5^. The Red Sea, characterized by high salinity, elevated temperatures, oligotrophic conditions, and high endemism, represents a unique and underexplored ecosystem that fosters the production of bioactive metabolites with distinct chemical signatures^6^.

*Stylissa carteri* is a common Red Sea sponge noted for its richness in secondary metabolites, particularly pyrrole–imidazole alkaloids, terpenoids, and peptides^7^. These compounds display potent antibacterial, antiviral, and cytotoxic activities, often acting by disrupting DNA synthesis, microtubule formation, or signaling pathways^8^. The species’ chemical diversity is partly attributed to its microbial symbionts, making it a promising source for anticancer drug discovery^9^. Similarly, *Hemimycale arabica*, endemic to the Red Sea, produces phenolic compounds, brominated alkaloids, and bioactive peptides with selective cytotoxicity against cancer cells^10^. Its metabolites can trigger apoptosis, activate caspases, and inhibit angiogenesis^11^. Environmental factors such as temperature and salinity influence its metabolite profile, yet it remains underexplored in detailed chemical–biological correlation studies^12,13^. Complementing these species, *Negombata magnifica* is a bright red sponge best known for producing latrunculins—macrolides that disrupt actin polymerization and show nanomolar cytotoxicity^14^. It also contains diterpenes, sterols, and alkaloids with strong anticancer potential^15^. Habitat variations in the Red Sea may affect its chemical composition and bioactivity, offering opportunities for targeted pharmacological exploration^16^.

Despite the recognized pharmacological potential of these species, most prior studies have focused on the isolation and structural elucidation of single metabolites, often without comprehensive integration of chemical profiling and functional bioactivity data^17^. Molecular docking and computational approaches have increasingly been used to predict drug–target interactions and guide drug discovery^18,19^, but their application to Red Sea sponge metabolites, particularly when combined with in vitro biological assays, remains limited. Comparative studies assessing how environmental variation influences metabolite diversity and biological potency in these sponges are also scarce.

To the best of our knowledge, this represents the inaugural study to concurrently examine the chemical diversity, as well as the phenolic and flavonoid content, and cytotoxicity of *S. carteri*, *H. arabica*, and *N. magnifica* collected from ecologically distinct Red Sea sites. By integrating LC–MS/MS-based chemical profiling with MTT cytotoxicity, colony formation, and wound healing assays, and validating bioactive candidates through molecular docking against Chk2 kinase, this work provides an integrated chemical–biological–computational framework. Moreover, it addresses the influence of environmental factors on secondary metabolite composition and bioactivity, offering new insight into the role of habitat variability in shaping the pharmacological potential of marine sponges. Therefore, this work provides an integrated chemical–biological–computational framework for the bioactivity-guided prioritization of sponge species and metabolites, and the generation of testable mechanistic hypotheses to guide subsequent preclinical development.

## Materials and methodology

### Sponge collection and identification

Specimens of three marine sponge species—*Hemimycale arabica*, *Stylissa carteri*, and *Negombata magnifica*—were collected from the Red Sea coastal region of Hurghada, Egypt. Sampling was conducted by SCUBA diving at depths of 10 to 18 m at three distinct sites: El Gouna, Abu Galawa, and Umm Gamar (southern Hurghada). Each species was collected in triplicate from different geographic locations to capture potential ecological and chemical variability. Immediately after collection, sponges were rinsed with sterile seawater to remove sand, epiphytes, and debris. Samples were placed in sterile, labeled plastic bags and transported on ice to the laboratory. Fresh tissue color and gross morphological features were recorded in situ and photographed underwater for documentation.

Taxonomic identification was conducted by Dr. Mohamed Ez El-Arab, sponge taxonomist at the National Institute of Oceanography and Fisheries (NIOF), Hurghada, Egypt. Species identification was based on external morphology and microscopic examination of skeletal spicules following standard protocols. Portions of each specimen were preserved in 70% ethanol for taxonomic reference and deposited in the NIOF sponge repository under voucher specimen numbers: Specimen No. 1 (*H. arabica*), No. 2 (*S. carteri*), and No. 3 (*N. magnifica*). All collections were performed under the authorization and in accordance with national regulations issued by the Egyptian Environmental Affairs Agency (EEAA). The study complied with local biodiversity conservation policies and ethical guidelines for marine bioprospecting.

### Extraction of the marine sponge material

The frozen marine sponge specimens (*Hemimycale arabica*, *Stylissa carteri*, and *Negombata magnifica*) were meticulously cut into small fragments and exhaustively extracted with a 1:1 (v/v) mixture of methanol (MeOH) and dichloromethane (CH_2_Cl_2_)^20^. Each sample underwent three consecutive extraction cycles of 48 h each to ensure maximal recovery of secondary metabolites. The combined MeOH/CH_2_Cl_2_ extracts were filtered and concentrated under reduced pressure at temperatures below 40 °C using a rotary evaporator (Rotavapor R-300, Büchi Labortechnik AG, Switzerland).

The resulting crude MeOH/CH_2_Cl_2_ extract was then suspended in distilled water and successively partitioned with solvents of increasing polarity—n-hexane, ethyl acetate (EtOAc), and n-butanol (n-BuOH)—to yield the corresponding solvent fractions. Each organic fraction was individually concentrated under reduced pressure, weighed, and stored at − 20 °C until further analysis.

### Evaluation of cytotoxicity

The total extracts and corresponding fractions derived from the selected marine sponges were evaluated for their potential cytotoxic activity using the human hepatocellular carcinoma cell line (HepG2). This investigation aimed to identify bioactive secondary metabolites with cytotoxic or antineoplastic properties. The human hepatocellular carcinoma cell line (HepG2) used in this study was obtained from Nawah Scientific Inc. (Cairo, Egypt). All extracts and fractions were initially dissolved in dimethyl sulfoxide (DMSO), sterilized using 0.22 µm syringe filters, and then diluted in complete Dulbecco’s Modified Eagle Medium (DMEM) to achieve the necessary working concentrations. The final concentration of DMSO in all treatments remained below 0.1% (v/v), which also served as the vehicle control. Doxorubicin (1 µM) was used as a positive control to benchmark the cytotoxicity of the sponge-derived extracts^21^. Extracts were tested in a concentration range of 1–200 µg/mL for cytotoxicity and colony formation assays. To comprehensively evaluate the antitumor effects, the following in vitro assays were employed:

#### MTT cell viability assay

The cytotoxicity of the sponge extracts and their fractions was investigated using the MTT assay, adhering to the methodology outlined by Mosmann^22^. HepG2 cells were cultured at a density of 1 × 10^4^ cells per well in 96-well plates and allowed to incubate overnight at 37°C in a humidified atmosphere with 5% CO_2_. The cells were then subjected to treatment for 24 h with different concentrations (1, 10, 25, 50, 100, and 200 µg/mL) of each extract. DMSO (0.1%) acted as the vehicle control, while doxorubicin (1 µM) was utilized as a positive control. After the treatment period, 20 µL of MTT solution (0.5 mg/mL) was added to each well and incubated for 3 h. The formazan crystals were dissolved using 150 µL of DMSO, and the absorbance was recorded at 590 nm with a microplate reader (Bio-Rad, USA). The percentage of viable cells relative to the untreated control was computed, and IC_50_ values were determined through nonlinear regression analysis.

#### Colony formation assay

The prolonged inhibitory impact of sponge extracts on the proliferation of HepG2 cells was evaluated utilizing the clonogenic assay, as outlined by Franken et al.^23^. Cells were plated in 6-well plates at a density of 200–300 cells per well and permitted to adhere overnight. Subsequently, the cells were treated for 72 h with extract concentrations of 10, 25, 50, and 100 µg/mL. Following the treatment, the medium was substituted with drug-free medium, and the cells were incubated for 10–15 days to facilitate colony development. Colonies (≥ 50 cells) were fixed using methanol, stained with 0.5% crystal violet, and counted under an inverted microscope. DMSO (0.1%) and doxorubicin (1 µM) were utilized as vehicle and positive controls, respectively. The results were presented as the percentage of colony formation in comparison to the control.

#### In vitro wound healing (scratch) assay

The inhibitory impact of sponge extracts on the migration of HepG2 cells was evaluated using a wound healing assay. Confluent monolayers of cells cultivated in 6-well plates were scratched with a sterile 200 µL pipette tip to create a consistent gap. The cells were carefully rinsed with PBS and subsequently treated with sub-cytotoxic concentrations of the extracts (generally 10 and 25 µg/mL, chosen based on MTT results) to minimize the contribution of cell proliferation to wound closure. Wound areas were captured at 0, 24, and 48 h using a phase-contrast microscope. Migration was assessed by calculating the percentage of wound closure relative to the initial scratch^24^. Since sub-cytotoxic concentrations were used, the observed wound closure is predominantly due to cell migration, rather than proliferation at the wound edg.

### Statistical analysis

All experiments were conducted independently in triplicate, and the results are expressed as mean ± standard deviation (SD). Statistical analysis was carried out using one-way ANOVA, followed by Tukey’s post hoc test. A *p*-value of less than 0.05 was deemed statistically significant.

### UPLC/ESI–MS profiling of the total MeOH:DCM extract of *Stylissa carteri*

A thorough metabolic profiling of the total MeOH:DCM extract of *S. carteri* was conducted using Ultra-Performance Liquid Chromatography in conjunction with Electrospray Ionization Mass Spectrometry (UPLC/ESI–MS). In summary, 1 mg of the extract was dissolved in 1 mL of HPLC-grade methanol (CH₃OH; Fisher Scientific, UK) and subsequently filtered through a 0.22 μm PTFE membrane filter (Sartorius, USA) before analysis.

Chromatographic separation was conducted using a Shimadzu Prominence HPLC system equipped with LC-20AD pumps and an SPD-M20A diode array detector (DAD), which was coupled to a Bruker amaZon speed ion trap mass spectrometer. Data acquisition was performed in positive ESI mode during a single run, with background subtraction implemented using a blank mobile phase. Aliquots (1 μL) of each prepared sample were injected onto an InertSustain C18 column (3.0 μm, 4.6 × 150 mm; GL Sciences, Japan). The mobile phase was composed of solvent A (water with 0.1% acetic acid) and solvent B (acetonitrile with 0.1% acetic acid). The elution gradient was set as follows: 10% B for 1 min, followed by a linear increase to 100% B over the subsequent 14 min, maintaining a constant flow rate of 0.4 mL/min^25^.

The ESI source parameters were as follows: capillary temperature, 320 °C; source voltage, 3.5 kV; sheath gas flow rate, 11 L/min. Mass spectra were acquired in full-scan mode over the m/z range 100–2000. For MSⁿ analysis, the ion charge control (ICC) target was set at 50,000, with a maximum acquisition time of 200 ms, a minimum threshold of 1000 counts, and a scan rate of 6 scans/s. Raw MS/MS data (*.d) were converted to mzML format using MSConvert from the ProteoWizard software suite (https://www.proteowizard.org).

### Molecular docking study

#### System preparation and molecular docking

The crystal structure of human checkpoint kinase 2 (Chk2) in complex with a debromohymenialdisine analog (PDB ID: 2CN8, resolution: 1.70 Å) was obtained from the Protein Data Bank^26^. The protein structure was prepared using UCSF Chimera^27^. The co-crystallized ligand (2-({4-[(2,4-dichlorobenzyl)oxy]phenyl}amino)-7,8-dihydro-6H-cyclohepta[d]pyrimidin-4(3H)-one, DBQ) and all water molecules were removed. Hydrogen atoms were added, and the protein structure was optimized at pH 7.5 using the PROPKA tool^28^.

*Identification of the active site*: The ATP-binding site (active site) of Chk2 was defined based on the binding location of the co-crystallized inhibitor (DBQ) in the 2CN8 structure. This site is a well-characterized, hydrophobic pocket located in the kinase hinge region between the N- and C-lobes, consistent with literature on Chk2 kinase inhibition^26^. The centroid of the DBQ ligand’s coordinates was used to define the center of the docking grid.

Molecular docking was performed using AutoDock Vina. The 2D structures of the test compounds (hymenialdisine, spongiacidin D, etc.) were drawn in ChemBioDraw Ultra 12.1 and converted to 3D, followed by energy minimization using the MMFF94 force field in Avogadro software. For docking, Gasteiger partial charges were assigned. A grid box of dimensions 20 × 20 × 20 Å^3^ was constructed, centered on the coordinates X =  − 10.85, Y = 43.78, Z = 8.3 Å, which encompassed the entire ATP-binding site as defined by the native ligand. An exhaustiveness value of 8 was used for the search. The docked conformations were generated using the Lamarckian Genetic Algorithm and ranked based on their binding energy scores (kcal/mol). The docking protocol was validated by redocking the native ligand (DBQ) into the defined grid, which yielded a satisfactory root-mean-square deviation (RMSD) of 0.80 Å between the docked and crystal poses.

#### Molecular dynamics (MD) simulations

To further explore the stability of ligand–protein complexes, molecular dynamics (MD) simulations were performed utilizing the GPU-accelerated PMEMD engine within the AMBER 18 package^29^. Atomic partial charges were assigned using the General Amber Force Field (GAFF) through the ANTECHAMBER module^27^. Each system was solvated in an orthorhombic box filled with TIP3P water molecules, maintaining a 10 Å buffer distance from the solute, and was neutralized by the addition of Na⁺ and Cl^−^ counterions via the Leap module.

Energy minimization was executed in two phases: an initial 2000 steps with a restraint potential of 500 kcal/mol, followed by 1000 steps of unrestrained minimization employing the conjugate gradient algorithm. The systems were then gradually heated from 0 to 300 K over a period of 500 ps, while keeping the atom count and volume constant. A harmonic restraint of 10 kcal/mol was applied to the solute atoms with a collision frequency of 1 ps. Following this, equilibration was performed for 500 ps under constant temperature (300 K) conditions. Production simulations were carried out under an isothermal–isobaric (NPT) ensemble, maintaining a pressure of 1 bar with the Berendsen barostat^30^. Each simulation lasted for 100 ns with a timestep of 2 fs. Bond lengths involving hydrogen atoms were constrained using the SHAKE algorithm. The temperature was controlled at 300 K with a Langevin thermostat set at a collision frequency of 1 ps, while pressure coupling was configured to 2 ps.

#### Post-MD analysis

Trajectory files produced during the molecular dynamics simulations were stored at 1 ps intervals and evaluated utilizing the CPPTRAJ module of the AMBER18 suite. The conformational stability and interaction dynamics of the protein–ligand complexes were investigated, and all graphical representations and molecular visualizations were created using Origin and UCSF Chimera^29^.

#### Thermodynamic calculations

Ligand–protein binding affinities were estimated using the molecular mechanics Poisson–Boltzmann surface area (MM/PBSA) and generalized Born surface area (MM/GBSA) methodologies, which are widely applied to evaluate free binding energies in biomolecular systems^31^. Binding free energy values were averaged from 250 snapshots extracted across the final 100 ns of the simulation trajectory.

The calculated free energy was decomposed into gas-phase energy, solvation free energy, and entropic contributions. Gas-phase energy included bonded, electrostatic, and van der Waals interactions, which were determined using the FF14SB force field parameters. Solvation contributions were divided into polar and non-polar components. The polar term was estimated using the generalized Born model, while the non-polar solvation energy was derived from the solvent-accessible surface area (SASA), applying a water probe radius of 1.4 Å. In addition, per-residue free energy decomposition was performed through the MM/GBSA protocol in AMBER18, which enabled the identification of key amino acid residues contributing to the stabilization of the ligand within the binding pocket^32,33^.

#### Pharmacophore mapping and in silico ADMET prediction

Pharmacophore Mapping for Target Identification: To predict potential protein targets for the lead metabolites identified by LC–MS/MS, a reverse pharmacophore mapping approach was employed. The 3D structures of hymenialdisine and spongiacidin D were energy-minimized and submitted to the PharmMapper server (http://www.lilab-ecust.cn/pharmmapper/)^34^. This server matches the input ligand’s pharmacophoric features against a database of receptor-based pharmacophore models derived from the PDB. The results were ranked by the fit score, and the human checkpoint kinase 2 (Chk2, PDB ID: 2CN8) was identified as a high-probability target, guiding the subsequent molecular docking studies.

In Silico* ADMET profiling*: The absorption, distribution, metabolism, excretion, and toxicity (ADMET) properties of the lead compounds were predicted using two established online platforms to assess their drug-likeness and potential pharmacokinetic issues.

- SwissADME (http://www.swissadme.ch)^35^ was used to predict key physicochemical and pharmacokinetic parameters. This included calculation of molecular weight (MW), number of hydrogen bond donors/acceptors (nHDon/nHAc), topological polar surface area (TPSA), consensus Log P (lipophilicity), and water solubility (Log S). Compliance with major drug-likeness rules (Lipinski, Ghose, Veber, Egan, Muegge) was evaluated. The bioavailability radar was generated to visually assess drug-likeness.
- The pkCSM platform (https://biosig.lab.uq.edu.au/pkcsm/)^36^ was used for toxicity prediction. Specific endpoints assessed included: AMES mutagenicity, inhibition of the hERG I/II channels (cardiotoxicity potential), hepatotoxicity, and skin sensitization.

## Results

### Extraction yield of marine sponge fractions

The total methanol–dichloromethane (1:1, v/v) extracts of *Hemimycale arabica*, *Stylissa carteri*, and *Negombata magnifica* were successively partitioned using solvents of increasing polarity—n-hexane, ethyl acetate (EtOAc), and n-butanol (n-BuOH)—to obtain distinct solvent fractions. The extraction procedure was conducted in three successive cycles to ensure maximum metabolite recovery. The yields of each fraction, calculated as the percentage of the total crude extract dry weight, are summarized in Table 1.

**Table 1:** Extraction yields (% w/w of total crude extract) of solvent fractions from *H. arabica*, *S. carteri*, and *N. magnifica*.

Sponge species	n-Hexane fraction (%)	EtOAc fraction (%)	n-BuOH fraction (%)
*Hemimycale arabica*	11.94 ± 0.6^cB^	16.21 ± 0.9^bB^	22.13 ± 1.1^aB^
*Stylissa carteri*	10.63 ± 0.5^ cc^	13.98 ± 0.8^bc^	19.56 ± 0.9^ac^
*Negombata magnifica*	14.00 ± 0.7^cA^	16.95 ± 1.0^bA^	22.14 ± 1.2^aA^

Values are expressed as mean ± standard deviation (n = 3) and calculated as the percentage of each fraction’s dry weight relative to the total crude extract. Solvent partitioning was performed sequentially using n-hexane, ethyl acetate (EtOAc), and n-butanol (n-BuOH). Different superscript letters (a–c) within the same row indicate statistically significant differences among solvent fractions (*p* < 0.05, one-way ANOVA followed by Tukey’s post hoc test).

Statistical analysis revealed significant differences in extraction yields both within and between sponge species (*p* < 0.05). Within each species, the *n*-butanol fraction exhibited the highest yield (denoted by “a”), followed by the ethyl acetate (“b”) and *n*-hexane (“c”) fractions. Across species, *N. magnifica* displayed significantly higher yields (denoted by “A”) in all solvent fractions compared with *H. arabica* (“B”) and *S. carteri* (“C”). These results confirm that both solvent polarity and species-specific metabolite profiles strongly influence extraction efficiency.

The yield differences among fractions and species likely reflect variations in secondary metabolite polarity and abundance. The *n*-butanol fractions yielded the highest percentages in all species (22.13 ± 1.1^aB^, 19.56 ± 0.9^ac^, and 22.14 ± 1.2^aA^ for *H. arabica*, *S. carteri*, and *N. magnifica*, respectively), indicating enrichment in polar constituents such as amino acids, peptides, glycosides, and sulfated metabolites that are typically abundant in marine sponges^37,38^. The ethyl acetate fractions (16.21 ± 0.9^bB^, 13.98 ± 0.8^bc^, and 16.95 ± 1.0^bA^) contained semi-polar compounds including phenolics, alkaloids, and terpenoids, previously reported from *Hemimycale*, *Stylissa*, and *Negombata* species^39^. Conversely, the *n*-hexane fractions (11.94 ± 0.6^cB^, 10.63 ± 0.5^ cc^, and 14.00 ± 0.7^cA^) exhibited the lowest yields, consistent with extraction of non-polar metabolites such as fatty acids, sterols, and hydrocarbons^9^.

Among the three species, *N. magnifica* consistently yielded higher extractable content across all solvents, likely due to its richness in brominated alkaloids and polar pyrroloiminoquinone derivatives^40^. These trends align with prior studies demonstrating that sequential solvent partitioning enriches distinct classes of metabolites according to solvent polarity^3^. A comparative visualization of extraction yields and statistical groupings is shown in Figure S1.

### Antitumor activity

#### Cytotoxicity assessment (MTT assay)

**Fig. 1:**
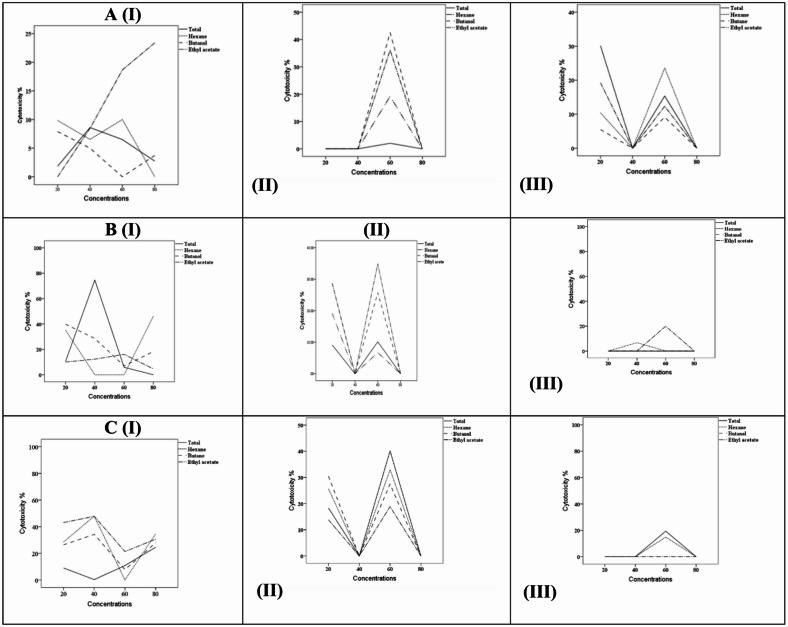
Cytotoxicity of total extracts and solvent fractions of marine sponges* (Stylissa carteri*, *Hemimycale arabica*, and *Negombata magnifica)* collected from three Red Sea sites (El Gouna, Abu Galawa, and Umm Gamar) against HepG2 cells as determined by the MTT assay. Data are presented as mean ± SD (n = 3). It should be noted that cytotoxicity was assessed over six concentrations (1–200 µg/mL), but only the mid-range (20–80 µg/mL) is presented for clarity. Some extracts showed mild deviations from dose-dependence, likely reflecting the complex nature of the crude mixtures and potential antagonistic or synergistic effects among bioactive compounds.

The cytotoxic effects of total extracts and solvent fractions derived from sponges were assessed in vitro against HepG2 human liver carcinoma cells utilizing the MTT assay. Samples were obtained from three different marine locations along the southern Red Sea coast of Egypt: El Gouna, Abu Galawa, and Umm Gamar (southern Hurghada). The extracts were evaluated at a concentration of 100 µg/mL, with cytotoxicity reported as the percentage of inhibition compared to untreated controls. As detailed in Table 2 and depicted in Fig. 1, the cytotoxic activity exhibited significant variation based on sponge species, solvent fraction, and geographical origin.

**Table 2:** Cytotoxicity (%) of sponge total extracts and solvent fractions at 100 µg/mL against HepG2 cells (MTT assay, mean ± SD, n = 3).

Sponge species	Location	Total extract	n-Hexane	Ethyl acetate	n-Butanol	IC_50_ (µg/mL)
*N. magnifica*	El Gouna	< 20	< 20%	< 20%	< 20%	–
	Abu Galawa	< 20	30 ± 3.5	< 20%	< 20%	–
	Umm Gamar	< 20	40 ± 4.2	< 20%	35 ± 3.8	–
*S. carteri*	El Gouna	80 ± 2.5	< 20%	< 20%	< 20%	37
	Abu Galawa	< 10	< 20%	< 20	35 ± 2.9	–
	Umm Gamar	< 20	< 20%	< 20	< 20%	–
*H. arabica*	El Gouna	< 20	45 ± 4.0	< 20	45 ± 3.5	–
	Abu Galawa	40 ± 3.8	30 ± 3.2	< 20	< 20%	–
	Umm Gamar	< 20	< 20	< 20	< 20%	–

Results (mean ± standard deviation ) obtained after 24 h treatment with 100 µg/mL of each extract or fraction.*Values represent percentage inhibition. Non-cytotoxic:* < *20% inhibition. Bolded values indicate moderate to high cytotoxicity (*≥ *30%).* Only the total extract of *Stylissa carteri* from El Gouna showed high cytotoxicity with an IC_50_ value of 37 µg/mL. All other samples showed low or moderate cytotoxicity and were not subjected to IC_50_ determination. Irregular dose–response patterns observed in some fractions likely reflect the complex mixture of metabolites, differences in solubility, stability, or potential synergistic/antagonistic interactions.

For *Negombata magnifica*, all fractions obtained from El Gouna demonstrated non-cytotoxic properties, with inhibition values remaining under 20%. In contrast, from Abu Galawa, only the n-hexane fraction displayed moderate activity at 30%, while the remaining fractions were non-cytotoxic. The most significant activity was noted in samples from Umm Gamar, where the n-hexane and n-butanol fractions showed cytotoxicity levels of 40% and 35%, respectively.

Regarding *Stylissa carteri*, samples from El Gouna indicated considerable activity in the total extract, which exhibited 80% cytotoxicity and an IC_50_ value of 37 µg/mL, while all fractions were below 20%. From Abu Galawa, the n-butanol fraction revealed moderate activity at 35%, whereas the total extract and other fractions showed minimal cytotoxicity. Samples from Umm Gamar exhibited consistently low activity across all extracts and fractions. The pronounced cytotoxic activity observed for the total MeOH:DCM extract of S. carteri from El Gouna, compared with its solvent-partitioned fractions, likely results from synergistic interactions among multiple bioactive metabolites. Fractionation separates compounds based on polarity, which can disrupt these synergistic effects and reduce the overall potency. Additionally, some active metabolites may be present in sub-effective concentrations in individual fractions or distributed across different fractions, further diminishing their cytotoxic impact. Thus, the total extract reflects the combined or additive effects of multiple metabolites, which may be required to achieve significant cytotoxicity against HepG2 cells.

Extracts of *Hemimycale arabica* from El Gouna exhibited significant activity, with both the n-hexane and n-butanol fractions demonstrating 45% cytotoxicity. From Abu Galawa, the total extract and n-hexane fraction displayed moderate cytotoxicity (40% and 30%, respectively), while other fractions remained below 20%. All fractions from Umm Gamar showed weak activity (< 20%). These findings indicate that both the chemical composition of the solvent extract and the site of collection have a considerable impact on the observed cytotoxic activity. Notably, *Stylissa carteri* from El Gouna, along with *Hemimycale arabica* from both El Gouna and Abu Galawa, exhibited the most promising cytotoxic potential and warrant further exploration.

It should be noted that the cytotoxicity assay was originally performed at six concentrations (1, 10, 25, 50, 100, and 200 µg/mL), as described in the Experimental Section; however, only the mid-range concentrations (20–80 µg/mL) are shown in Fig. 1 to enhance visual clarity and focus on the biologically active region where measurable cytotoxicity was detected. Although some extracts exhibited non-linear responses across concentrations, such deviations from a strict dose-dependent pattern are not uncommon in crude extracts due to the combined effects of multiple constituents with differing solubility, stability, and bioactivity profiles.

Some extracts, particularly crude or partially fractionated samples, displayed irregular or non-monotonic dose–response trends. Such deviations may arise from the complex nature of crude extracts, including the presence of multiple metabolites with differing solubility, stability, or synergistic/antagonistic interactions. These factors can produce fluctuations in measured cytotoxicity, and are not uncommon in natural product studies. Therefore, IC_50_ values and cytotoxic trends should be interpreted with caution, especially for crude extracts, as the observed effects reflect the combined activity of multiple constituents rather than a single pure compound.

#### Colony formation assay

To evaluate the long-term antiproliferative effects of sponge extracts and their solvent fractions, a colony formation assay was performed on HepG2 cells that were treated with specific samples at a concentration of 50 µg/mL for a duration of 72 h. After the treatment, the cells were placed in drug-free medium for a period of 10 to 15 days to facilitate colony regrowth. Doxorubicin (1 µM) was utilized as a positive control, whereas untreated cells served as the negative control. The findings are presented in Table 3. The total MeOH:DCM extract of *Stylissa carteri* from El Gouna significantly suppressed colony formation, reducing the surviving fraction to 20.3 ± 2.1%, closely matching the effect of doxorubicin (15.2 ± 1.8%, *p* < 0.01). However, its solvent fractions (n-hexane, ethyl acetate, and n-butanol) showed no significant effect, with survival rates exceeding 55%.

**Table 3:** Effect of sponge total extracts and fractions on HepG2 colony formation at 50 µg/mL.

Sponge Species	Location	Extract	Surviving Fraction (%)	SD	Significance vs. Control
		Untreated Control	100.0	3.1	—
		Doxorubicin (1 µM)	15.2	1.8	*p* < 0.01
*Stylissa carteri*	El Gouna	total MeOH:DCM extract	20.3	2.1	*p* < 0.01
		n-Hexane	58.4	3.5	ns
		Ethyl Acetate	63.1	2.9	ns
		n-Butanol	55.7	3.2	ns
	Abu Galawa	total MeOH:DCM extract	88.6	3.8	ns
		n-Hexane	66.4	2.7	ns
		Ethyl Acetate	70.2	3.4	ns
		n-Butanol	*42.5*	2.8	*p* < *0.05*
	Umm Gamar	total MeOH:DCM extract	73.4	4.0	ns
		n-Hexane	68.1	2.6	ns
		Ethyl Acetate	71.6	2.9	ns
		n-Butanol	69.3	3.1	ns
*Hemimycale arabica*	El Gouna	total MeOH:DCM extract	62.8	3.6	ns
		n-Hexane	30.7	2.4	*p* < 0.01
		Ethyl Acetate	59.2	3.3	ns
		n-Butanol	34.8	2.6	*p* < *0.01*
	Abu Galawa	total MeOH:DCM extract	*40.9*	3.0	*p* < *0.05*
		n-Hexane	*45.3*	2.7	*p* < *0.05*
		Ethyl Acetate	61.7	2.8	ns
		n-Butanol	57.5	2.9	ns
	Umm Gamar	total MeOH:DCM extract	76.2	3.9	ns
		n-Hexane	71.4	2.5	ns
		Ethyl Acetate	73.6	2.7	ns
		n-Butanol	70.9	3.0	ns
*Negombata magnifica*	El Gouna	total MeOH:DCM extract	81.5	3.2	ns
		n-Hexane	79.4	2.6	ns
		Ethyl Acetate	83.1	3.0	ns
		n-Butanol	80.3	3.4	ns
	Abu Galawa	total MeOH:DCM extract	79.6	3.1	ns
		n-Hexane	*61.3*	2.9	*p* < *0.05*
		Ethyl Acetate	76.7	3.2	ns
		n-Butanol	74.5	3.0	ns
	Umm Gamar	total MeOH:DCM extract	70.1	2.8	ns
		n-Hexane	*62.3*	3.2	*p* < *0.05*
		Ethyl Acetate	68.4	3.0	ns
		n-Butanol	66.1	2.9	*p* < *0.05*

Data represent mean percentage of colony survival ± standard deviation (n = 3). Statistical significance determined by one-way ANOVA followed by Tukey’s post hoc test. Bold: strong antiproliferative effect (≤ 35% survival). *Italic*: moderate inhibition (36–60% survival). *p* < 0.01: highly significant; *p* < *0.05*: significant; ns: not significant vs. untreated control.

At the Abu Galawa site, *S. carteri*'s total extract and most fractions were weakly active, except for the n-butanol fraction, which moderately inhibited colony formation (42.5 ± 2.8%, *p* < 0.05). Extracts from Umm Gamar were largely inactive, with all survival values above 68%, indicating limited long-term cytostatic or cytotoxic effects.

For *Hemimycale arabica*, samples from El Gouna were the most bioactive. Both the n-hexane and n-butanol fractions exhibited pronounced antiproliferative effects, with surviving fractions of 30.7 ± 2.4% and 34.8 ± 2.6%, respectively (*p* < 0.01). The ethyl acetate and total extracts were weakly to moderately active. Extracts from Abu Galawa also showed moderate effects; the total extract (40.9 ± 3.0%) and n-hexane fraction (45.3 ± 2.7%) significantly, though modestly, reduced colony formation (*p* < 0.05). In contrast, all extracts from Umm Gamar exhibited minimal activity, with colony survival remaining above 70%.

For *Negombata magnifica*, the overall antiproliferative activity was low. Extracts from El Gouna and Abu Galawa had minor impact, with surviving fractions mostly exceeding 74%. Only the n-hexane and n-butanol fractions from Umm Gamar showed moderate reductions in colony formation (62.3 ± 3.2% and 66.1 ± 2.9%, respectively; *p* < 0.05), though these effects were still relatively weak compared with the other sponges. These results collectively indicate that species type, solvent polarity, and collection site influence long-term growth inhibition, with *Stylissa carteri* (El Gouna) and *Hemimycale arabica* (El Gouna and Abu Galawa) showing the most consistent antiproliferative activity.

The observed loss of antiproliferative and anti-migratory activities in the solvent-partitioned fractions of *Stylissa carteri* compared to its total MeOH:DCM extract can be attributed to several factors. The total extract likely contains a synergistic combination of polar and semi-polar metabolites, that collectively enhance its biological activity. During fractionation, this synergistic balance is often disrupted, and individual fractions may contain lower concentrations of active metabolites or lack essential co-factors that potentiate cytotoxic effects. Moreover, some bioactive constituents could be distributed across multiple solvent layers in sub-effective amounts, resulting in diminished overall potency. Therefore, the pronounced activity of the total MeOH:DCM extract reflects the combined or additive effects of multiple metabolites, rather than the action of a single compound class.

#### Wound healing (scratch) assay

To evaluate the anti-migratory potential of sponge extracts, a wound healing assay was conducted using HepG2 cells. Sub-cytotoxic concentrations (25 µg/mL) of total MeOH:DCM extracts and solvent fractions (n-hexane, ethyl acetate, and n-butanol) were tested for each sponge species collected from El Gouna, Abu Galawa, and Umm Gamar. Doxorubicin (1 µM) was used as a positive control, and untreated cells served as the negative control. The wound closure percentage after 24 h was used as an indicator of cell migration. Results are presented in Table 4. Among all tested samples, the total MeOH:DCM extracts of *Stylissa carteri* from El Gouna demonstrated the strongest inhibition of cell migration, resulting in 35.4 ± 2.1% wound closure after 24 h, compared to 85.3 ± 2.7% in the control and 30.2 ± 1.9% in the doxorubicin-treated group (p < 0.01). Its solvent fractions showed limited anti-migratory activity.

**Table 4:** Inhibition of HepG2 cell migration by sponge extracts after 24 h (Wound Healing Assay at 25 µg/mL).

Sponge species	Location	Extract	Wound closure (%)	SD	Significance vs. control
		Untreated Control	85.3	2.7	—
		Doxorubicin (1 µM)	30.2	1.9	*p* < *0.01*
*Stylissa carteri*	El Gouna	Total MeOH:DCM extract	35.4	2.1	*p* < *0.01*
		n-Hexane	67.8	3.0	ns
		Ethyl Acetate	70.6	2.9	ns
		n-Butanol	66.2	2.6	ns
	Abu Galawa	total MeOH:DCM extract	75.5	2.5	ns
		n-Hexane	72.3	2.7	ns
		Ethyl Acetate	74.2	3.0	ns
		n-Butanol	62.1	2.8	*p* < *0.05*
	Umm Gamar	Total MeOH:DCM extract	78.9	2.6	ns
		n-Hexane	75.8	3.1	ns
		Ethyl Acetate	79.2	2.9	ns
		n-Butanol	76.5	2.7	ns
*Hemimycale arabica*	El Gouna	Total MeOH:DCM extract	55.4	2.4	*p* < *0.05*
		n-Hexane	45.1	2.3	*p* < *0.01*
		Ethyl Acetate	60.6	2.5	*p* < *0.05*
		n-Butanol	49.3	2.6	*p* < *0.01*
	Abu Galawa	Total MeOH:DCM extract	*59.8*	3.2	*p* < *0.05*
		n-Hexane	63.5	2.9	ns
		Ethyl Acetate	66.7	2.7	ns
		n-Butanol	60.4	2.6	*p* < *0.05*
	Umm Gamar	Total MeOH:DCM extract	74.3	2.8	ns
		n-Hexane	70.2	3.0	ns
		Ethyl Acetate	72.5	3.1	ns
		n-Butanol	71.6	2.8	ns
*Negombata magnifica*	El Gouna	Total MeOH:DCM extract	77.9	3.0	ns
		n-Hexane	76.4	2.9	ns
		Ethyl Acetate	79.5	2.6	ns
		n-Butanol	75.8	3.2	ns
	Abu Galawa	Total MeOH:DCM extract	76.6	2.7	ns
		n-Hexane	73.1	2.5	ns
		Ethyl Acetate	74.4	2.8	ns
		n-Butanol	70.2	2.6	ns
	Umm Gamar	total MeOH:DCM extract	71.7	2.9	ns
		n-Hexane	*65.7*	2.5	*p* < *0.05*
		Ethyl Acetate	69.1	2.7	ns
		n-Butanol	*68.1*	2.8	*p* < *0.05*

Values represent the percentage of wound closure after 24 h (mean ± SD, n = 3). *p* < 0.01 = highly significant; *p* < *0.05* = significant; ns = not significant vs. untreated control. Bold: strong migration inhibition (≤ 50% wound closure) . *Italic*: moderate migration inhibition (51–65%).

For *Hemimycale arabica*, both the n-hexane and n-butanol fractions from El Gouna significantly suppressed cell motility, with 45.1 ± 2.3% and 49.3 ± 2.6% closure, respectively (*p* < 0.01). Moderate inhibition was observed in the total extract from Abu Galawa (59.8 ± 3.2%, *p* < 0.05), while all extracts from Umm Gamar displayed minimal activity (wound closure > 70%).

In contrast, *Negombata magnifica* extracts had weak effects on cell migration. Only the n-hexane and n-butanol fractions from Umm Gamar moderately reduced wound closure to 65.7 ± 2.5% and 68.1 ± 2.8%, respectively (*p* < *0.05*), whereas all other fractions exhibited migration comparable to the untreated control. These results indicate that El Gouna-derived extracts, particularly from *Stylissa carteri* and *Hemimycale arabica*, possess the most promising anti-migratory potential, supporting their possible role in limiting tumor metastasis.

Based on the in vitro antitumor assays, *Stylissa carteri*, particularly the total MeOH:DCM extract collected from El Gouna, demonstrated the most potent and consistent cytotoxic activity among the three investigated sponge species. This extract exhibited 80% inhibition in the MTT assay at 100 µg/mL with an IC_50_ value of 37 µg/mL, significantly reduced HepG2 colony formation to 20.3 ± 2.1%, and markedly suppressed cell migration in the wound healing assay with only 35.4 ± 2.1% wound closure after 24 h—results comparable to the standard chemotherapeutic agent doxorubicin. In contrast, while *Hemimycale arabica* (notably its n-hexane and n-butanol fractions from El Gouna) showed moderate activity, and *Negombata magnifica* exhibited only mild effects, their bioactivity profiles were less consistent and less potent. Therefore, the total MeOH:DCM extract of *Stylissa carteri* from El Gouna was selected for further phytochemical investigation, including quantitative and qualitative analysis of its phenolic constituents via LC–MS to identify potential bioactive metabolites responsible for its observed cytotoxic effects.

### LC–MS/MS-based putative identification of metabolites in *Stylissa carteri* extracts

The total MeOH:DCM extracts of *Stylissa carteri* collected from three different Red Sea locations (El Gouna, Abu Galawa, and Umm Gamar) were analyzed using LC–MS/MS to compare their chemical profiles. This is the first report, to the best of our knowledge, comparing the secondary metabolite composition of *S. carteri* across these distinct marine habitats. The analysis resulted in the tentative identification of five pyrrole-imidazole alkaloids—a class of marine sponge-specific bioactive compounds—as well as one phenazine derivative, as detailed in Table 5. Compound identification was based on comparison of precursor and product ion spectra (MS/MS) with literature-reported fragmentation data for *Stylissa*-derived alkaloids and the Competitive Fragmentation Modeling for Metabolite Identification database (CFM-ID).

**Table 5:** Tentatively identified compounds in the total MeOH:DCM extract of *Stylissa carteri* from three Red Sea locations based on LC–MS/MS analysis (positive mode).

Peak	Rt (min.)	Mono *m/z*	[M + H]^+^	[M + NH_4_]^+^	[M + Na]^+^	MS/MS fragments	Tentative identification	Formula	Source	El Gouna	Abu Galawa	Umm Gamar
1	1.2	245.0912	246.0991	263.1250	268.0810	217, 229	*E-*debromohymenialdisine	C_11_H_11_N_5_O_2_	PubChem/Cayman/MedChem	+ +	–	+ +
2	1.8	304.9912	305.9990	323.0250	327.9809	202,254, 295,297, 307,281, 239	Hymenialdisine	C_11_H_8_BrN_5_O	PubChem	+ +	+ + +	+
3	2.1	323.9858	324.9936	342.0196	346.9755	254,227, 202,307, 297, 282	Spongiacidin D	C_11_H_9_BrN_4_O_3_	PubChem	+	+ + +	+ +
4	2.8	309.0225	310.0303	327.0563	332.0122	312,122, 293, 295	Hymenidin	C_11_H_12_BrN_5_O	PubChem	–	+	–
5	3.5	386.9330	387.9408	404.9668	409.9228	122,139, 371	Oroidin	C_11_H_11_Br_2_N_5_O	PubChem	+	+ +	_
6	29.1	311.0906	312.0984	329.1244	334.0803	295,284, 268	Dermacozine H	C_16_H_13_N_3_O_4_	NPASS/ChemSpider	+	+	–

Identifications are based on comparison of retention time (*Rt)*, precursor ion (M⁺), and fragment ion data with literature and CFM-ID predictions. ( +) = detected; (+ + / +  + +) = relative abundance.

**Fig. 2:**
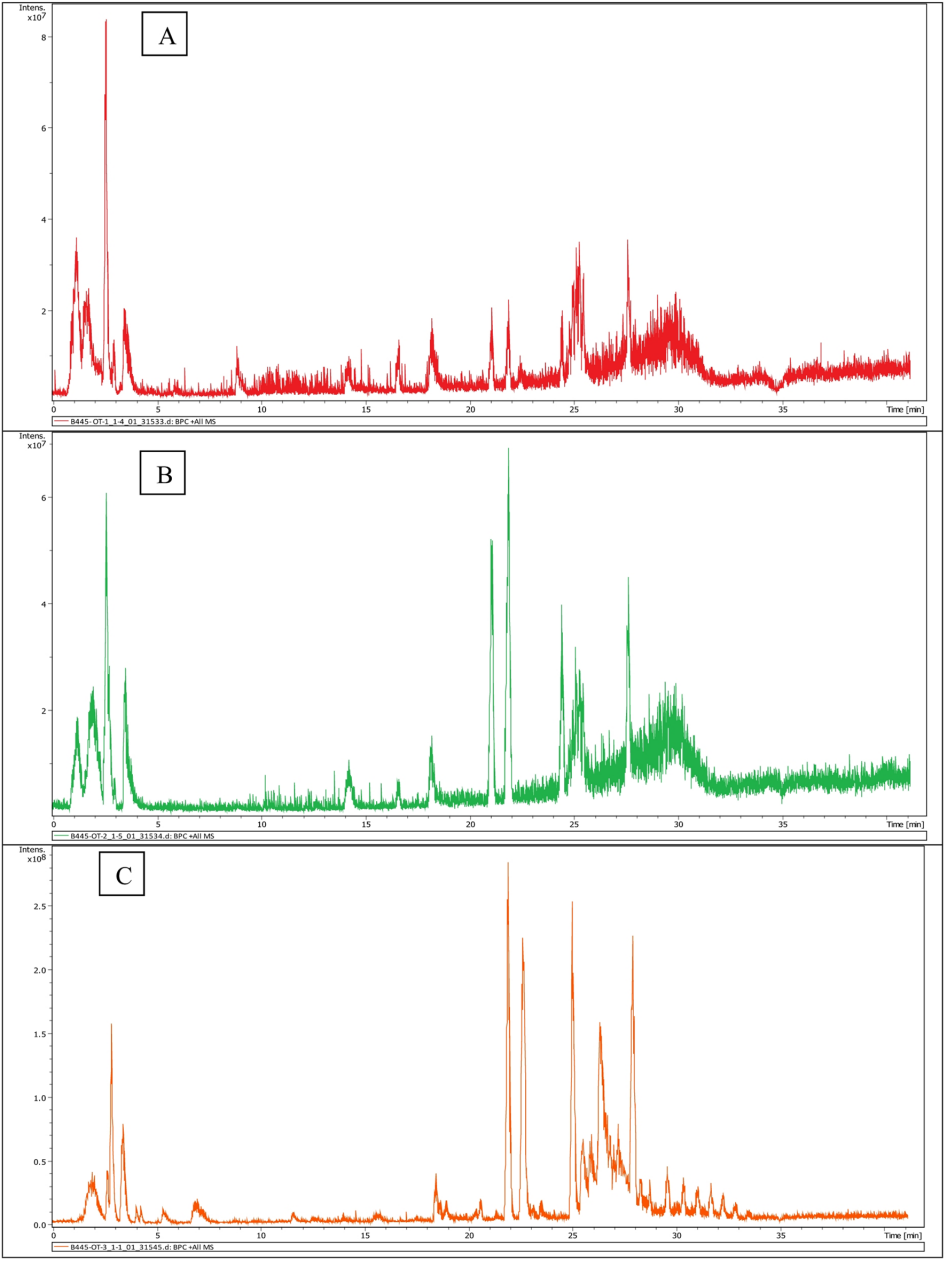
Comparative base peak chromatograms (BPCs) obtained by ESI–MS/MS for total MeOH:DCM extracts of *Stylissa carteri* collected from (**A**) El Gouna, (**B**) Abu Galawa, and (**C**) Umm Gamar.

The LC–MS spectra revealed characteristic bromine isotope patterns, including 1:1 intensity for mono-brominated and 1:2:1 for di-brominated pyrrole-imidazole alkaloids, supporting the identification of brominated alkaloid structures. A comparative base peak chromatogram (Fig. 2) showed site-specific differences in compound distribution. At Location 1 (El Gouna), the dominant compounds were hymenialdisine, oroidin, and dermacozine H, with *E-*debromohymenialdisine being the most prominent. At Location 2 (Abu Galawa), the extract was rich in hymenialdisine, hymenidin, oroidin, spongiacidin *D*, and dermacozine H, with hymenialdisine, oroidin, and hymenidin being predominant. At Location 3 (Umm Gamar), the major identified compounds were *E*-debromohymenialdisine, spongiacidin D, and hymenialdisine, with spongiacidin D being the most abundant.

**Fig. 3:**
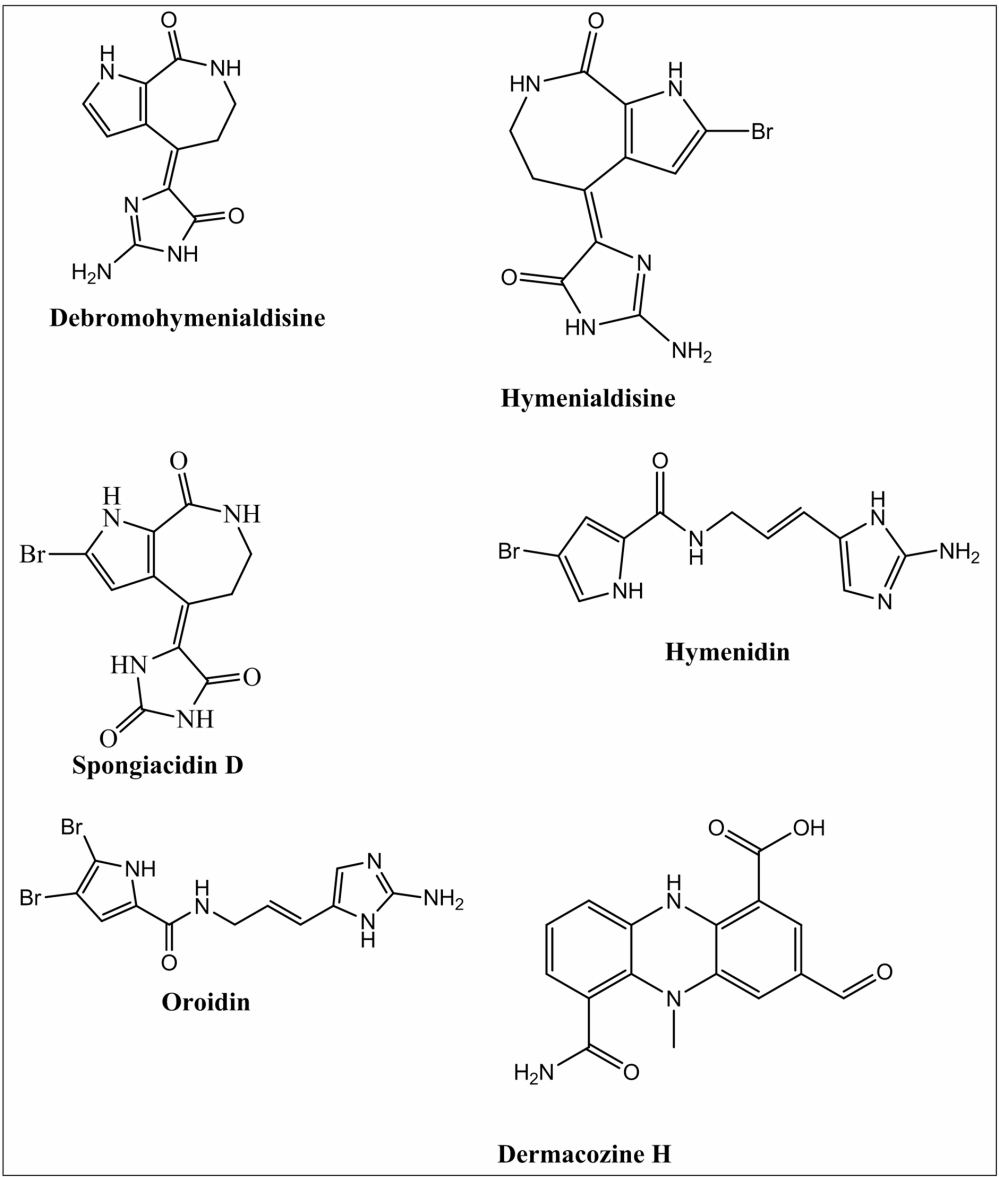
Chemical structure of the identified alkaloids in *Stylissa carteri.*

A comparative base peak chromatogram showed clear site-specific differences in compound distribution among the three locations. Notably, the total MeOH:DCM extract of *Stylissa carteri* collected from Umm Gamar exhibited a distinct chemical profile compared to those from El Gouna and Abu Galawa, with spongiacidin D and *E-*debromohymenialdisine being predominant, while other key alkaloids such as hymenidin and oroidin were absent or present only in trace amounts. These differences reflect habitat-related variations in secondary metabolite biosynthesis.It should be noted that these identifications are putative and based on MS/MS fragmentation and database matching; confirmation using authentic standards and NMR analysis will be required to validate these assignments. Chemical structure of the identified alkaloids is presented in Fig. 3.

These results demonstrate that *S. carteri* collected from El Gouna is chemically distinct and enriched in unique bioactive alkaloids, correlating with its superior in vitro cytotoxic activity. Therefore, this extract is an ideal candidate for further phytochemical and pharmacological exploration. The LC–MS/MS profiling of *Stylissa carteri* total MeOH:DCM extracts revealed a rich composition of brominated alkaloids and phenazine-type compounds, many of which have been previously reported as bioactive marine metabolites with significant pharmacological potential. The presence of pyrrole-imidazole alkaloids, such as hymenialdisine, oroidin, spongiacidin D, and hymenidin, is particularly noteworthy, as these compounds are known for their diverse bioactivities including cytotoxic, anti-inflammatory, and kinase-inhibitory properties. Notably, Hymenialdisine, prominent in both El Gouna and Abu Galawa samples, has been reported to inhibit cyclin-dependent kinases and induce apoptosis in cancer cells. Similarly, oroidin and spongiacidin D have been linked to antiproliferative effects via inhibition of cell cycle regulators and tumor invasion pathways. The exclusive detection of *E*-Debromohymenialdisine in El Gouna and Umm Gamar, and its prominence in El Gouna, may partially explain the superior cytotoxic and anti-migratory effects observed from the El Gouna extract. Additionally, the identification of Dermacozine H, a phenazine-like compound, suggests the presence of redox-active structures that could contribute to reactive oxygen species (ROS)-mediated cytotoxicity. Collectively, these findings provide a strong chemical basis for the observed antitumor activity and validate the selection of the El Gouna total MeOH:DCM extract of *S. carteri* for further identification, characterization, and mechanistic evaluation of its bioactive constituents. It should be noted that the identifications reported here are putative, based on MS/MS fragmentation patterns and database matching. Confirmation using authentic standards or NMR spectroscopy is necessary to unequivocally assign the structures.

### Molecular docking analysis

#### Pharmacophore mapping and molecular docking

To generate a mechanistic hypothesis for the potent anti-proliferative activity observed (e.g., suppression of colony formation), a reverse pharmacophore mapping screen was performed for the lead LC–MS/MS-identified metabolites, hymenialdisine and spongiacidin D. Using the PharmMapper server, both compounds were matched against a database of pharmacophore models derived from known protein targets. The top-ranked prediction identified the ATP-binding site of human checkpoint kinase 2 (Chk2; PDB ID: 2CN8) as a potential target. Chk2 is a key regulator of the DNA damage response and cell cycle arrest; its inhibition is a recognized strategy to impair cancer cell proliferation and survival, aligning with our phenotypic data^41^. Therefore, Chk2 was selected as a plausible and high-priority candidate target for computational evaluation of the identified metabolites’ binding potential.

#### Molecular docking validation

The molecular docking workflow was first validated by redocking the co-crystallized ligand (DBQ) into the Chk2 active site. This yielded a pose with a root-mean-square deviation (RMSD) of 0.80 Å relative to the crystal structure and a binding energy of − 11.30 kcal/mol, confirming the reliability of our docking parameters and grid definition. Among the identified sponge metabolites docked against Chk2, hymenialdisine demonstrated the most favorable binding affinity, with a docking score of − 8.35 kcal/mol. Spongiacidin D also showed a considerable affinity, with a score of − 7.30 kcal/mol. The detailed docking results for all tested compounds are provided in Table S1.

#### Molecular dynamic and system stability

**Fig. 4:**
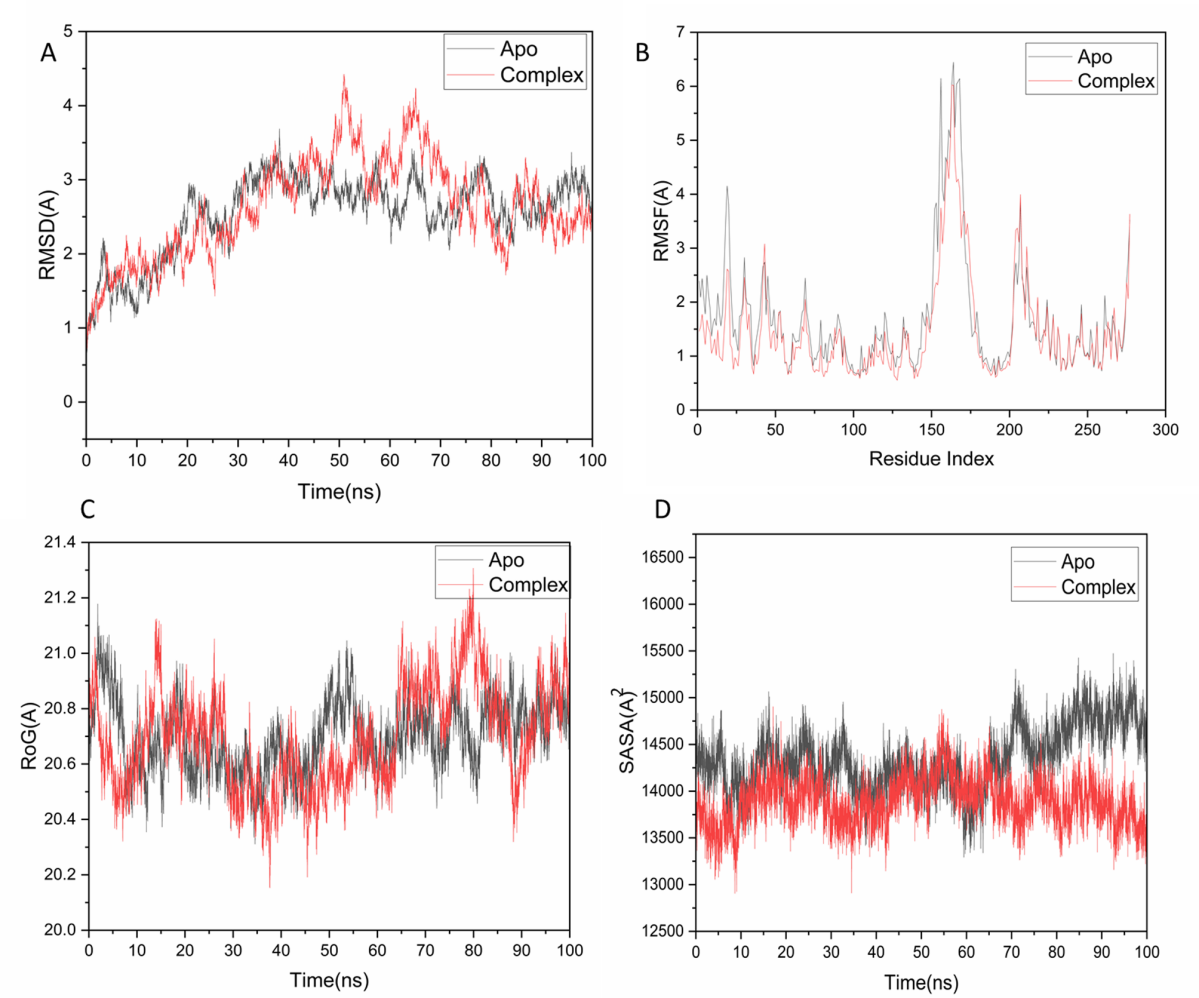
(**A**) RMSD of Cα atoms of the protein backbone atoms. (**B**) RMSF of each residue of the protein backbone Cα atoms of protein residues (**C**) ROG of Cα atoms of protein residues; (**D**) Solvent accessible surface area (SASA) of the Cα of the backbone atoms relative (black) to the starting minimized over 100 ns for the ATP binding site of human checkpoint kinase 2 receptor with Hymenialdisine (red).

A molecular dynamics (MD) simulation was performed to assess the binding efficiency of the identified compound with the active site of the target protein and to examine the stability of the complex throughout the simulation^42,43^. Assessing system stability is essential for identifying disrupted motions and avoiding artifacts that may occur during the simulation. This study examined the Root-Mean-Square Deviation (RMSD) to assess the stability of the systems throughout the 100 ns simulations. This study examined the Root-Mean-Square Deviation (RMSD) to assess the stability of the systems throughout the 100 ns simulations. The average RMSD values recorded for all frames of the systems were 2.65 ± 0.60 Å for the Apo and 2.54 ± 0.48 Å for the complex, as illustrated in Fig. 4A. The findings suggested that Hymenialdisine associated with the protein complex system achieved a relatively more stable conformation compared to the other systems analyzed. In molecular dynamics modeling, it is crucial to evaluate protein structural flexibility in response to ligand binding to analyze residue behavior and their interaction with the ligand^44^. Docking results for the identified compounds in *Stylissa carteri* total extract with the chk2 receptor are presented in Table S1.

The Root-Mean-Square Fluctuation (RMSF) technique was utilized to assess the fluctuations of protein residues and determine the impact of inhibitor binding on specific targets throughout 100 ns simulations. The average RMSF values calculated were 1.70 ± 0.9 Å for the Apo system and 1.46 ± 0.14 Å for the complex system, as shown in Fig. 4B. The findings indicated that the protein complex system bound to Hymenialdisine demonstrates reduced residue variation compared to the other systems. ROG -Radius of Gyration- was designed to evaluate the overall compactness and stability of the system upon ligand binding during molecular dynamics simulations^45,46^. The mean Rg values recorded were 20.81 ± 0.12 Å for the Apo system and 20.70 ± 0.17 Å for the complex system, as depicted in Fig. 4C.

Hymenialdisine exhibits an exceptionally rigid structure in relation to the target receptor. The density of the protein’s hydrophobic core was assessed by quantifying its solvent accessible surface area (SASA). This assessment involved measuring the surface area of the protein that is accessible to the solvent, which is crucial for the stability of biomolecules^47^. The average SASA values were 14,411.76 Å^2^ for the Apo system and 13,916.13 Å^2^ for the complex system, as depicted in Fig. 4D. The findings regarding SASA, in conjunction with the results from the RMSD, RMSF, and ROG analyses, validated that the Hymenialdisine complex system maintains stability within the catalytic domain binding site of the target receptor.

#### Binding interaction mechanism based on binding free energy calculation

The molecular mechanics/generalized Born surface area (MM/GBSA) method is extensively utilized for estimating the free binding energy of small molecules interacting with biomacromolecules, as it integrates both generalized Born and solvent-accessible surface area models, frequently yielding more dependable predictions than docking scores^48^. In this research, the MM/GBSA tool available in AMBER18 was employed to calculate binding free energies by obtaining representative snapshots from the simulation trajectories.

The binding energy components calculated for the Hymenialdisine–Chk2 complex are shown in Table 6. Except for the solvation free energy (ΔGsolv), all energy terms exhibited negative values, which suggests favorable interactions between Hymenialdisine and the protein’s catalytic binding site. The total binding free energy (ΔGbind) was measured at − 33.17 ± 0.17 kcal/mol, with significant contributions from van der Waals interactions (− 41.10 ± 0.50 kcal/mol), complemented by electrostatic interactions (− 7.29 ± 0.10 kcal/mol).

**Table 6:** Calculated binding energy components for Hymenialdisine against the catalytic binding site of human checkpoint kinase 2 receptor.

Energy components (kcal/mol)					
Complex	ΔE_vdW_	ΔE_elec_	ΔG_gas_	ΔG_solv_	ΔG_bind_
Hymenialdisine	− 41.10 ± 0.5	− 7.29 ± 0.10	− 48.39 ± 0.59	15.21 ± 0.21	− 33.17 ± 0.17

∆EvdW = van der Waals energy; ∆Eele = electrostatic energy; ∆Gsolv = solvation free energy; ∆Gbind = calculated total binding free energy.

#### Identification of the critical residues responsible for ligands binding

**Fig. 5:**
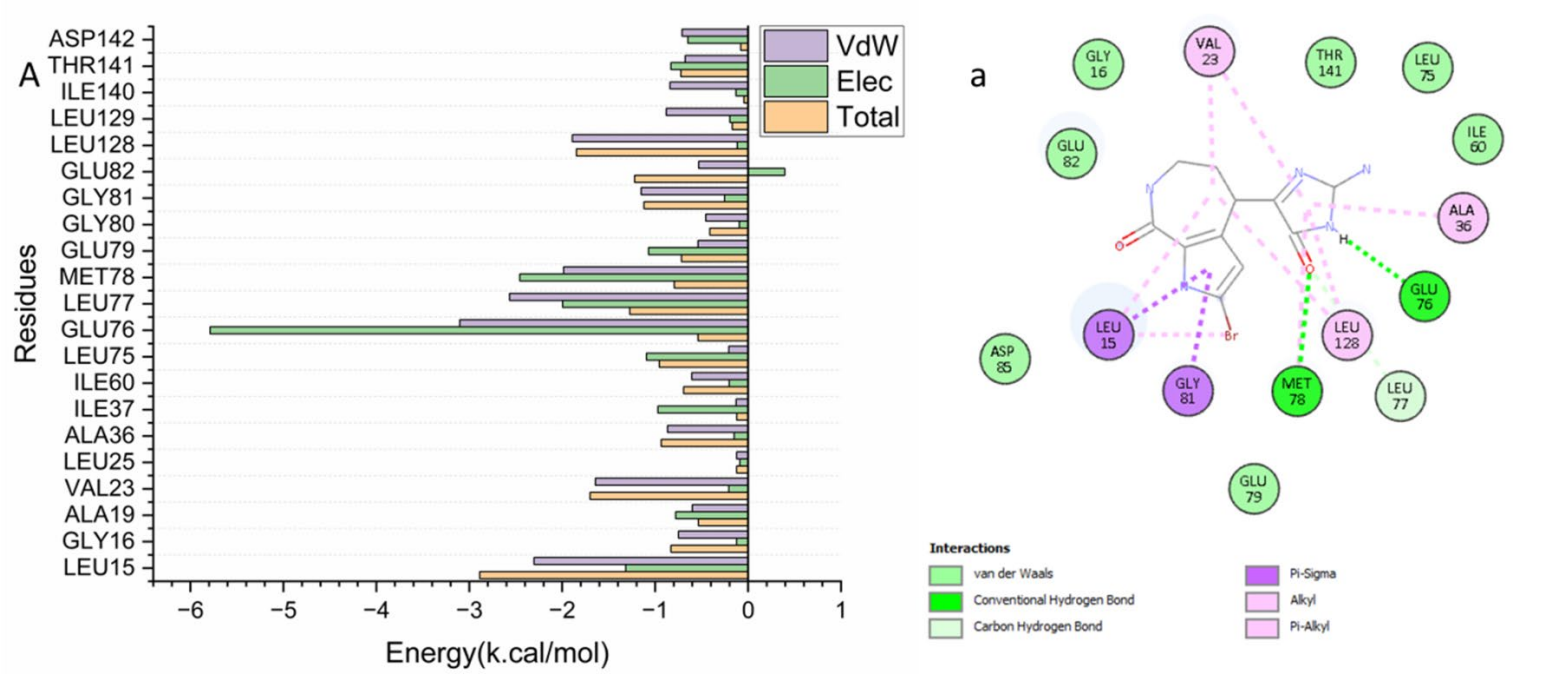
Per-residue decomposition plots showing the energy contributions to the binding and stabilization of Hymenialdisine within the ATP-binding site of the human checkpoint kinase 2 receptor.

To obtain additional insights into the molecular factors influencing inhibition, the binding free energy of Hymenialdisine with human checkpoint kinase 2 (Chk2) was analyzed by breaking it down into contributions from individual residues. As shown in Fig. 5, several residues located within the ATP-binding pocket were identified as having a significant impact on ligand stabilization. The most prominent contributions were observed from Leu15 (− 2.30 kcal/mol), Val23 (− 1.64 kcal/mol), Glu76 (− 3.10 kcal/mol), Leu77 (− 2.57 kcal/mol), Met78 (− 1.99 kcal/mol), and Leu128 (− 1.89 kcal/mol). These residues are considered crucial stabilizing hotspots within the catalytic site of Chk2.

#### Ligand–residue interaction network profiles

Ligand–residue interaction profiling was further performed to characterize the binding mechanism at the atomic level. In drug design, such analyses are fundamental for identifying specific structural features that enhance binding affinity, reduce toxicity, and optimize pharmacokinetic properties^49,50^. Drug action typically results from selective interactions between functional groups of a ligand and active site residues of the receptor, which subsequently initiate signal transduction events^51^.

**Fig. 6:**
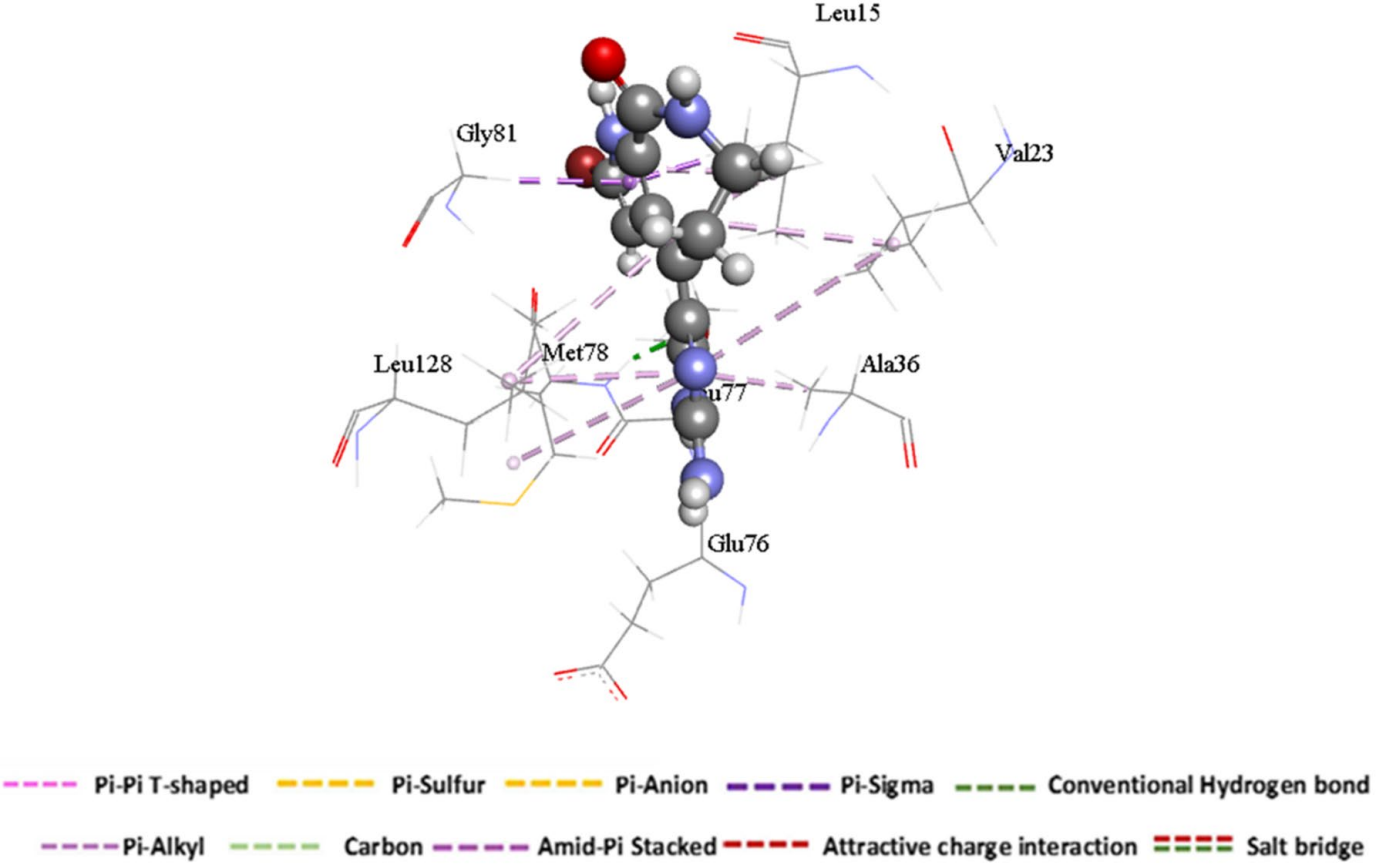
Molecular visualization of Hymenialdisine at the binding site residues of the human checkpoint kinase 2 receptor.

The analysis revealed that most active site residues of Chk2 were involved in hydrophobic interactions with Hymenialdisine. As shown in Fig. 6, residues Glu76 and Met78 established hydrogen bonds with the imidazole ring of the ligand, providing strong polar interactions. In addition, Val23 and Leu125 formed alkyl and π–alkyl interactions, while the hotspot residue Leu15 engaged in both π–sigma and π–alkyl interactions with Hymenialdisine. A further π–sigma interaction was observed between the ligand and Gly81, supporting the overall stability of the complex.

#### Principal component analysis (PCA)

**Fig. 7:**
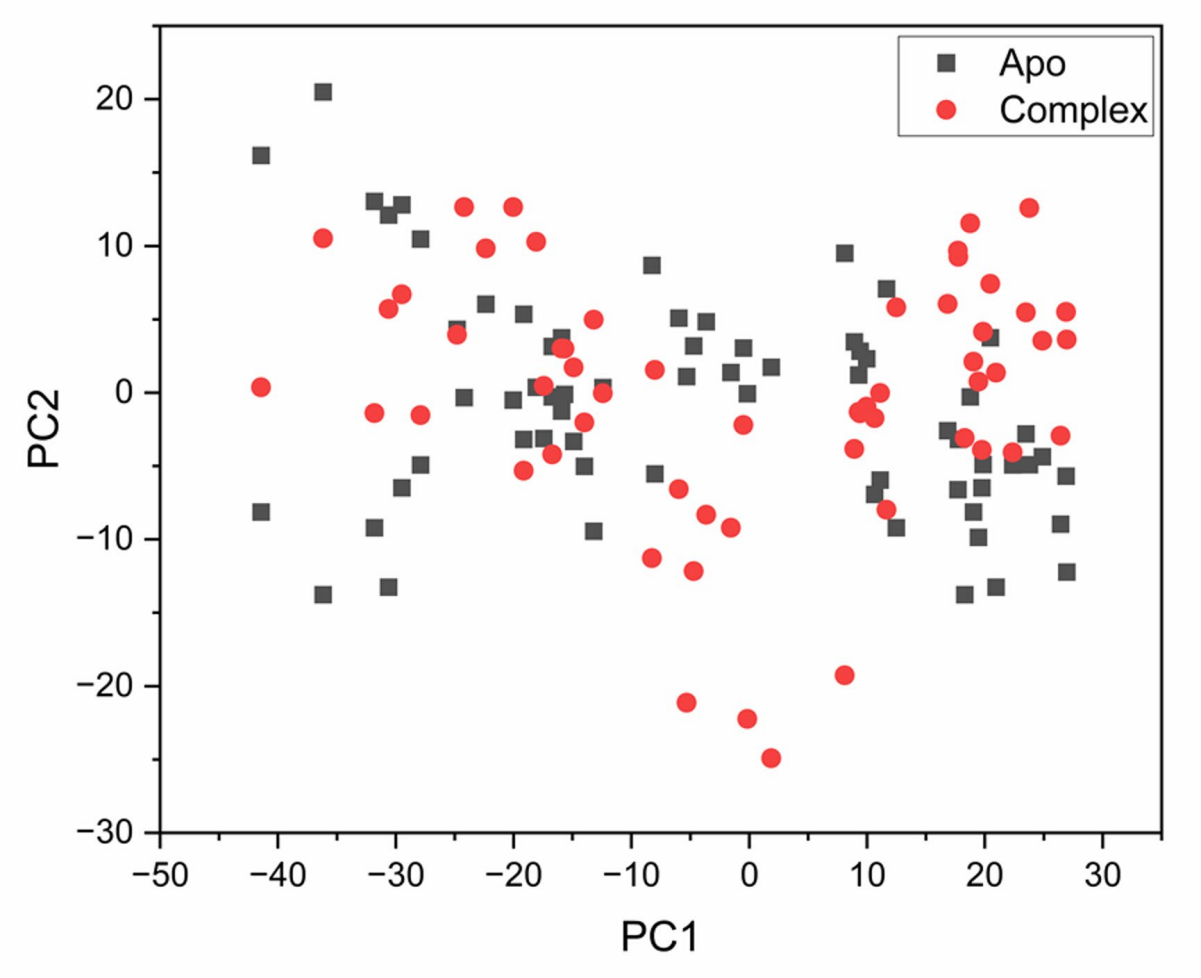
PCA projection of Cα atom motions constructed by plotting the first two principal components (PC1 and PC2) in conformational space for the apo system (black) and Hymenialdisine-complex (red) of the human checkpoint kinase 2 receptor.

Principal component analysis (PCA) was conducted to evaluate large-scale conformational motions of the apo-protein and the ligand-bound complex. The projection of the first two principal components is shown in Fig. 7. Distinct differences in motion were observed between the apo system and Hymenialdisine-bound system. The apo structure exhibited higher atomic fluctuations, while the Hymenialdisine–bound complex displayed reduced conformational variability. This suggests that ligand binding restricts the dynamic flexibility of the protein and stabilizes its catalytic domain.

#### Dynamics cross-correlation matrices (DCCM) analysis

**Fig. 8:**
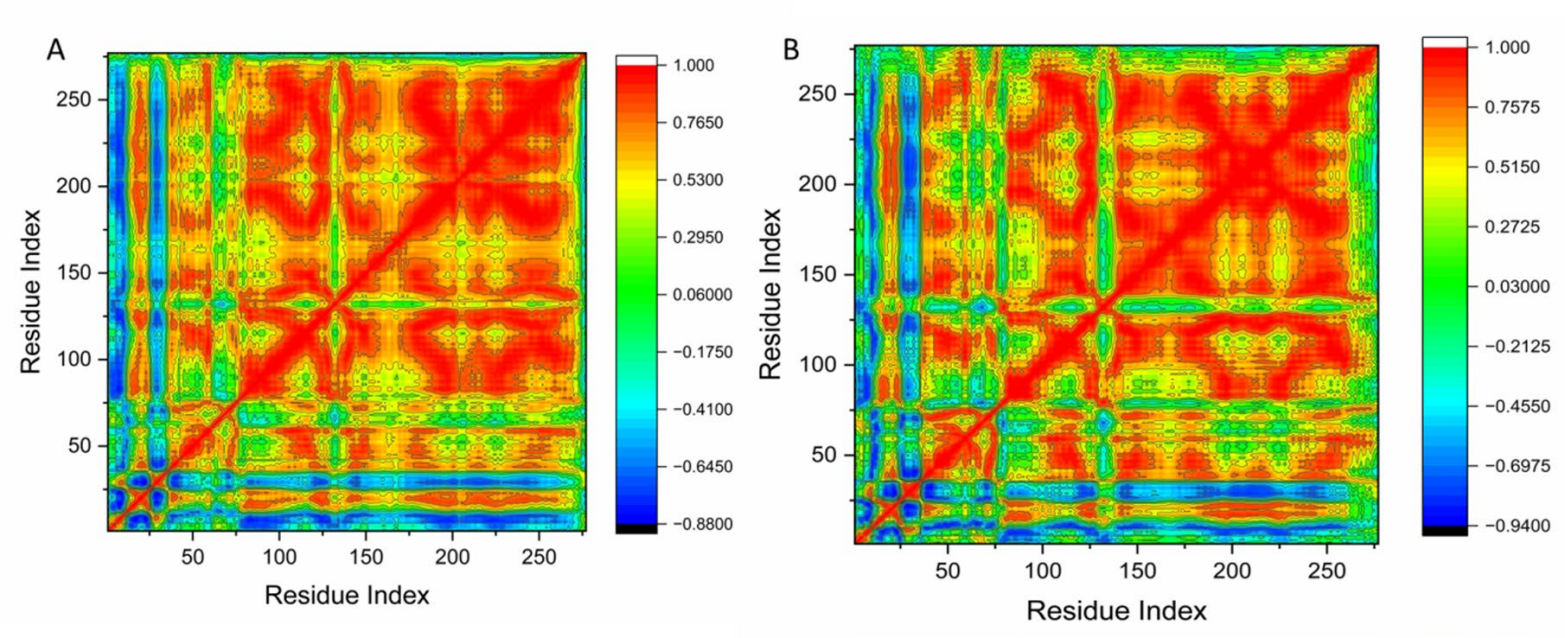
(**A**) Dynamic cross-correlation matrix analysis for Apo, and (**B**) Hymenialdisine binding to human checkpoint kinase 2 proteins. Numbers closer to 1 indicate high correlation, while those closer to –1 indicate anticorrelation between residue pairs.

During the simulations, DCCM analysis was carried out on the Cα positions to investigate the kinetics and coupled motions of residues and to assess the structural changes in the human checkpoint kinase 2 receptor upon ligand binding. Yellow–red regions represent strongly positive correlated motions, whereas blue–black regions indicate strongly anti-correlated motions. Overall, the analyzed systems displayed a predominance of correlated motions compared to anti-correlated ones. The interaction of Hymenialdisine with human checkpoint kinase 2 proteins revealed structural dynamics that induced conformational alterations, reflected as variations in residue correlation. Figure 8 highlights that the most correlated region lies between residues 150–250 of the checkpoint kinase 2 protein. These regions are highly dynamic and contain the majority of the hydrophobic active site residues.

#### Free energy landscape (FEL) analysis

**Fig. 9:**
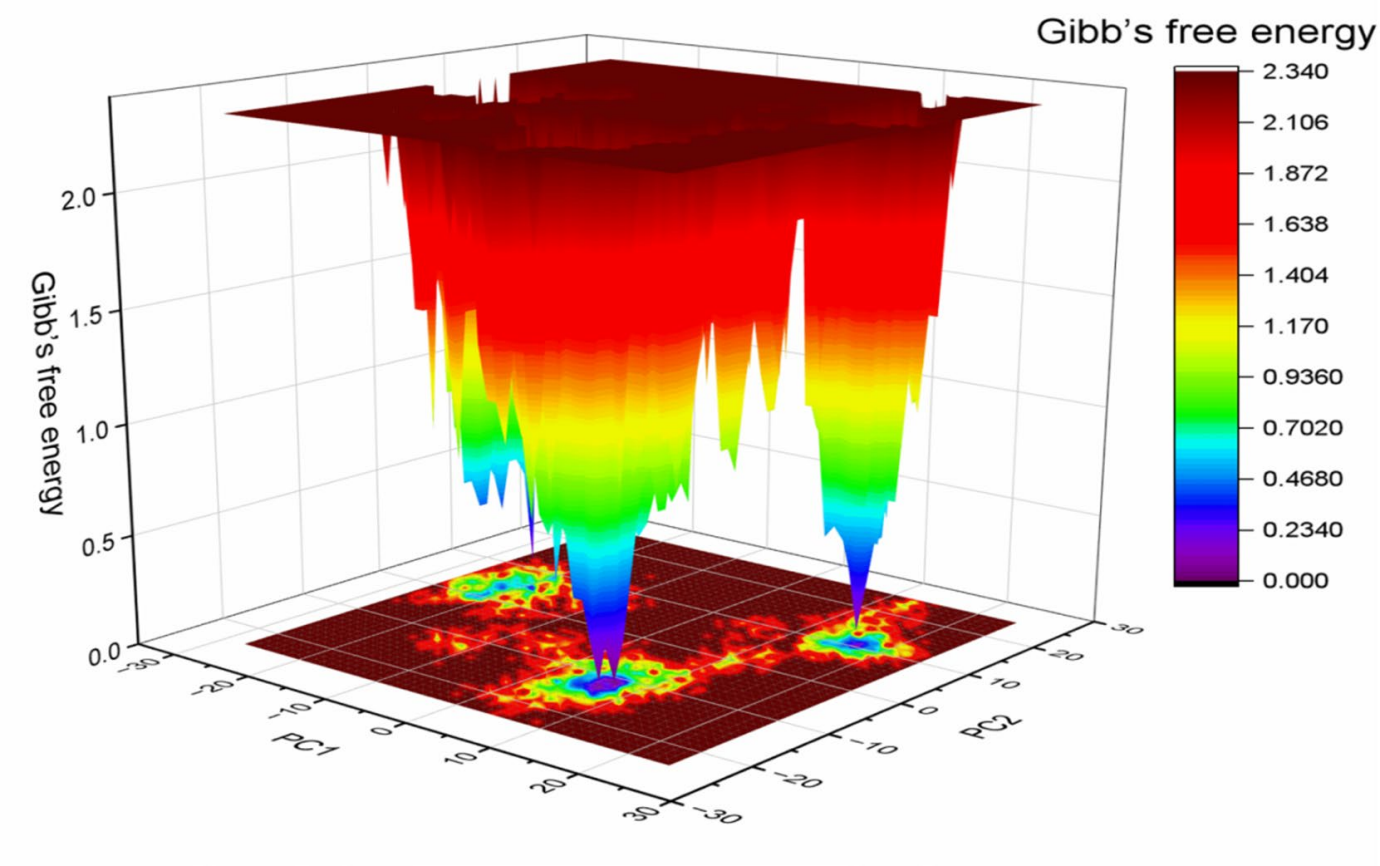
Free energy landscape (FEL) of the human checkpoint kinase 2 receptor– Hymenialdisine complex.

Protein conformational stability is associated with lower Gibbs free energy values; therefore, a PCA-based free energy landscape (FEL) analysis was performed to assess the stability of the simulated complexes. The FEL map revealed that the complex adopted stable conformations characterized by low Gibbs free energy states (blue and violet regions, Fig. 9).

#### Probability density function (PDF) analysis

**Fig. 10:**
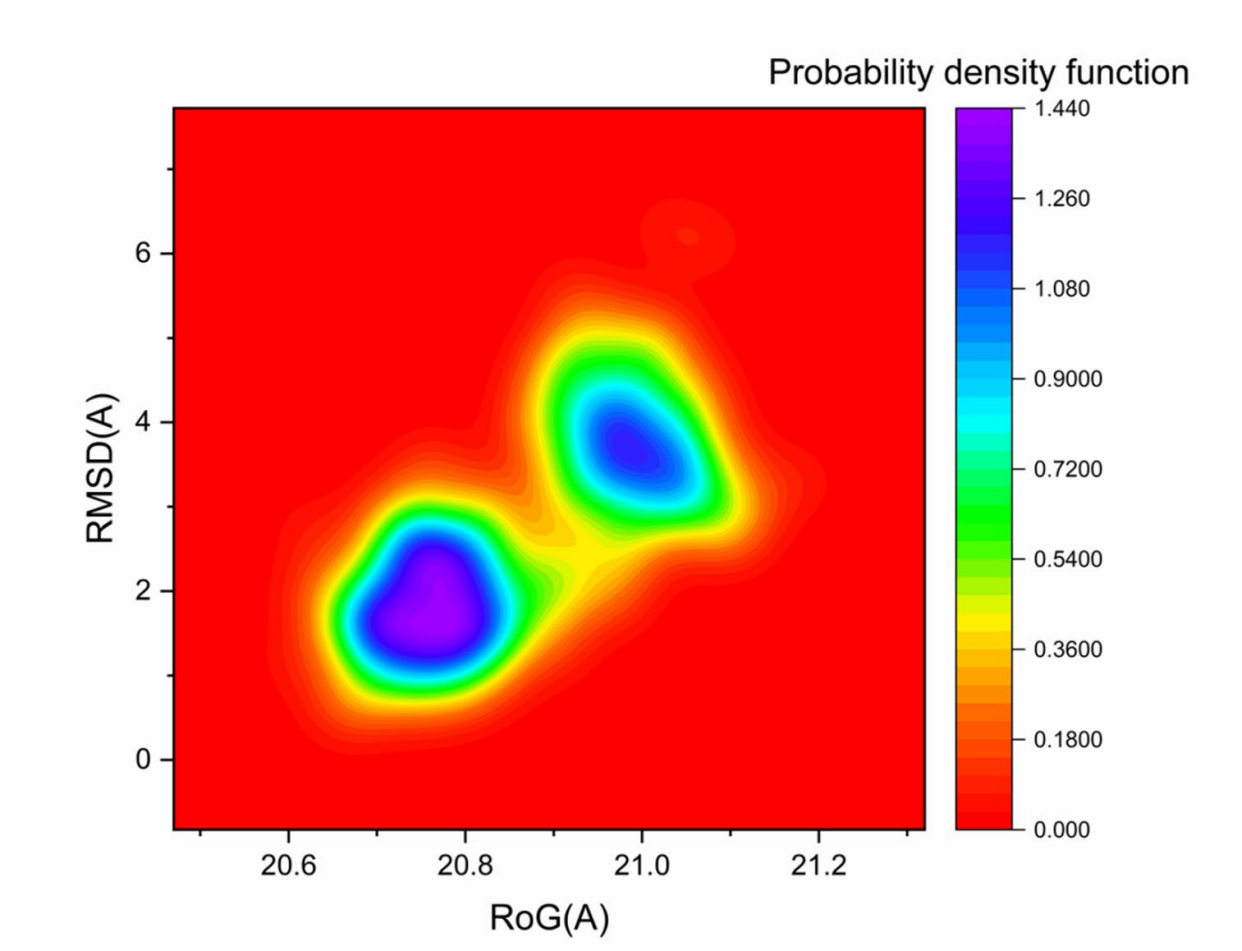
Probability density function (PDF) of the human checkpoint kinase 2– Hymenialdisine complex showing the least (red) and most (blue) populated conformations.

The probability density function (PDF) analysis provides the likelihood of protein trajectory distributions based on kernel density estimation (KDE)^52^. Figure 10 presents the PDF plots derived from the radius of gyration (Rg) and RMSD values for the human checkpoint kinase 2–Hymenialdisine complex. The analysis showed that the most densely populated conformations corresponded to an Rg value of 20.76 Å and an RMSD value of 1.76 Å.

#### In silico ADMET studies

The prediction of ADMET properties (absorption, distribution, metabolism, excretion, and toxicity) is a crucial step in drug discovery and development. ADMET profiling helps optimize the pharmacokinetics and overall drug-likeness of candidate molecules^53^. Here, the pharmacokinetic parameters of hymenialdisine and spongiacidin D were predicted using SwissADME^35^ and pkCSM^36^ and compared against established drug-likeness filters, including Lipinski, Veber, Egan, Ghose, and Muegge rules. Both compounds complied fully with Lipinski’s and Veber’s criteria^54^ (Table 7), indicating favorable oral bioavailability.

**Table 7:** ADME prediction of Hymenialdisine and Spongiacidin D by SwissADME.

Compound	Formula	MW	NHD	NHA	NRB	TPSA(A°)	MR	Fraction Csp3	XLOGP3
Hymenialdisine	C_11_H_10_BrN_5_O_2_	324.13	4	3	0	112.37	82.32	0.18	-0.16
Spongiacidin D	C_11_H_9_BrN_4_O_3_	325.12	4	3	0	103.09	79.91	0.18	0.16

Following the molecular docking screen, hymenialdisine was identified as the lead candidate due to its strong binding affinity for the Chk2 receptor (docking score: – 8.35 kcal/mol). To contextualize its drug-likeness and explore structure–property relationships, we compared its profile to spongiacidin D, a structurally similar compound from the same source that showed significantly weaker binding (docking score: – 7.30 kcal/mol).

**Fig. 11:**
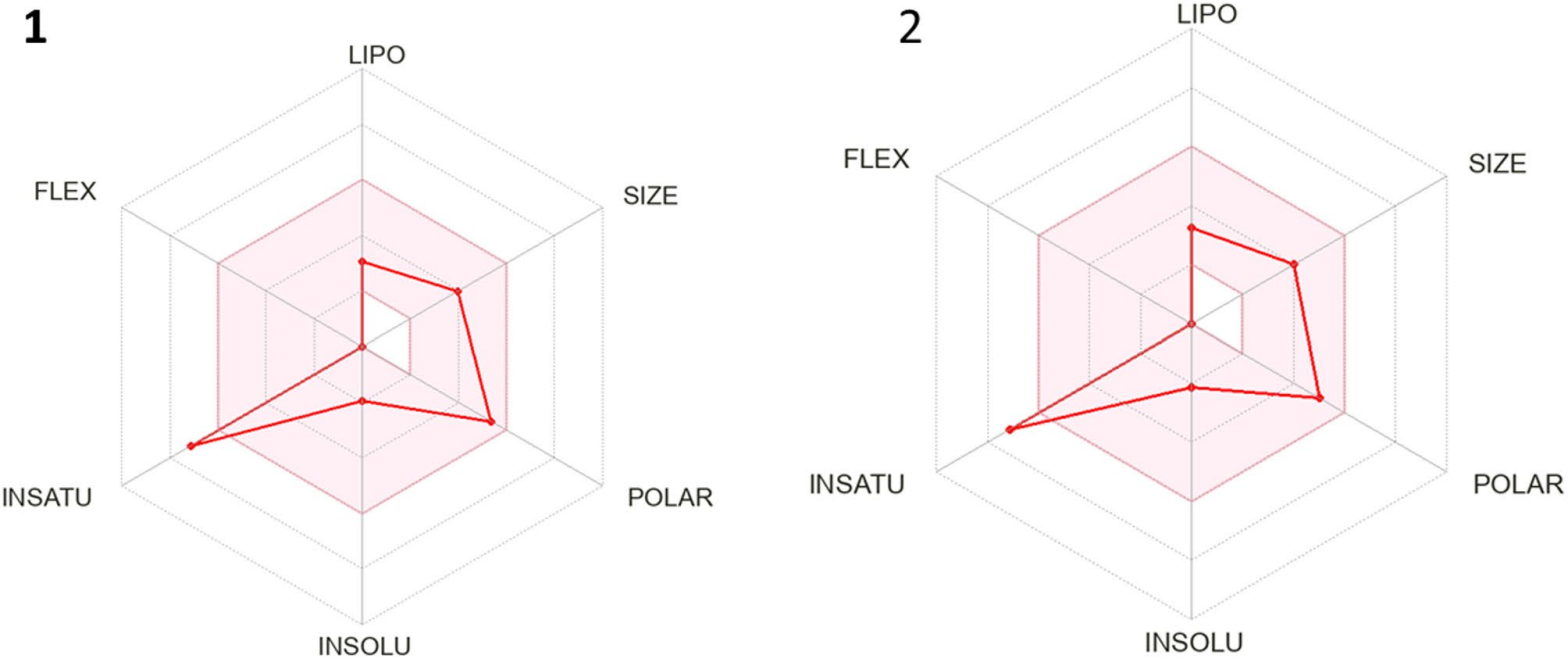
Bioavailability radar of hymenialdisine and spongiacidin D.

The bioavailability radar plots (Fig. 11) further illustrate the drug-likeness of hymenialdisine and spongiacidin D. The pink region represents the optimal range for key molecular properties: lipophilicity (XLOGP3: – 0.16 to + 0.16), molecular weight (324–325 g mol^−1^), polarity (TPSA: 103–112 Å^2^), solubility (log S ≤ 6), saturation (fraction Csp^3^ ≥ 0.25), and flexibility (≤ 9 rotatable bonds).

#### Solubility

**Fig. 12:**
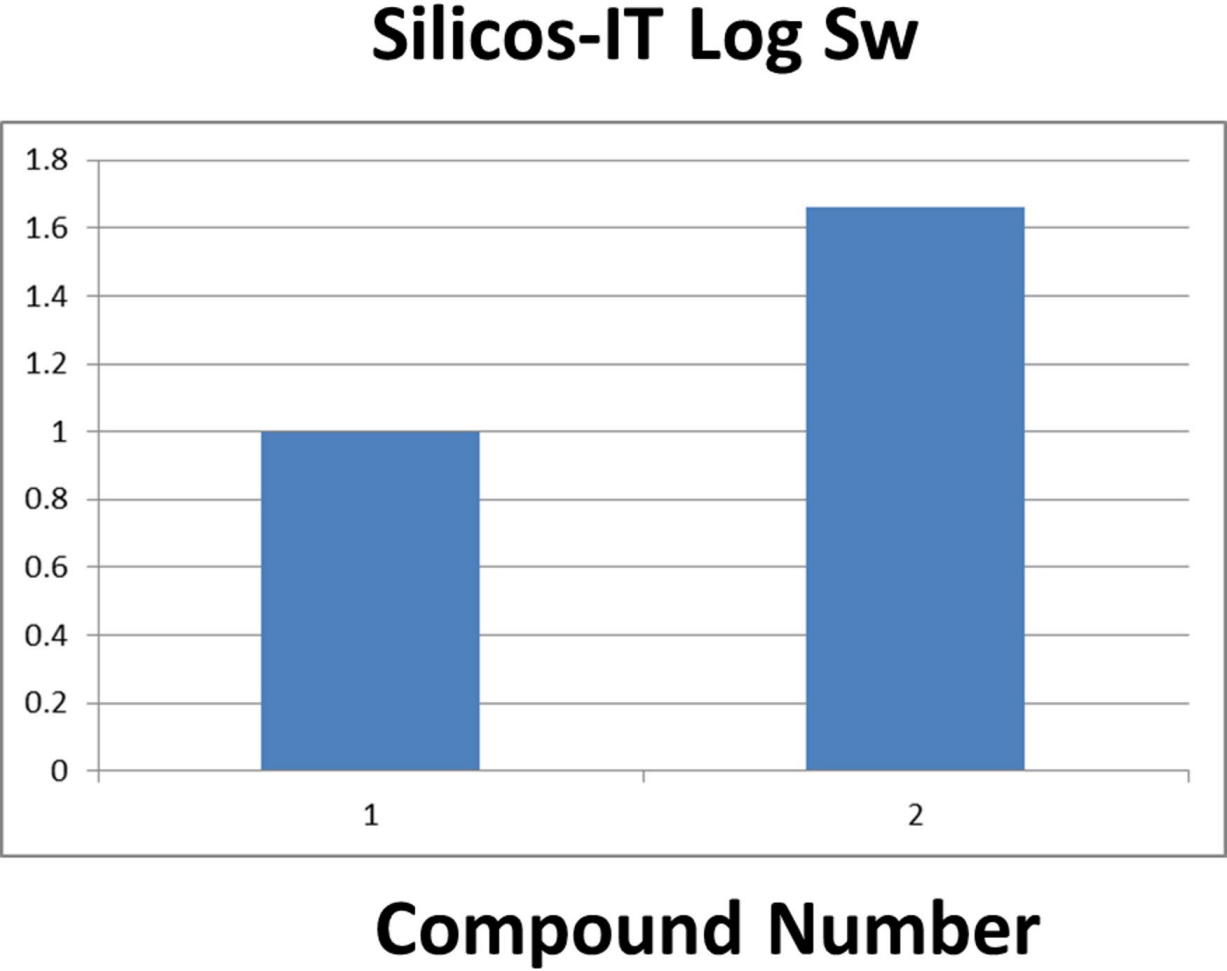
Log S values of hymenialdisine (1) and spongiacidin D (2).

Solubility in intestinal fluid is essential for efficient absorption and therapeutic efficacy^55^. SwissADME evaluates aqueous solubility through the Silicos-IT Log S parameter, which estimates the decimal logarithm of molar solubility in water. As shown in Fig. 12, hymenialdisine and spongiacidin D exhibit a balance between aqueous solubility and membrane permeability, critical for oral bioavailability. spongiacidin D showed slightly better water solubility than hymenialdisine, as indicated by its lower Log P value.

#### Lipophilicity

**Fig. 13:**
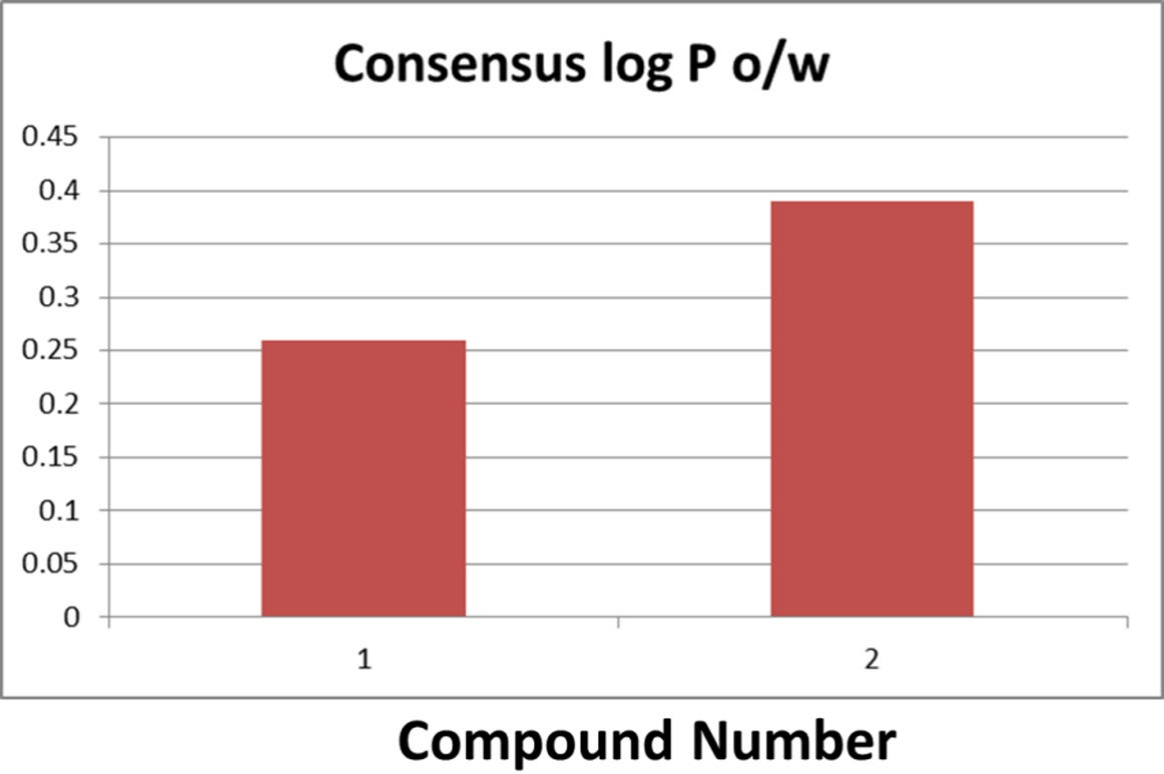
Consensus Log *P* values of hymenialdisine (1) and spongiacidin D (2).

Lipophilicity, expressed as the partition coefficient (log P o/w), reflects membrane permeability. Extremely low lipophilicity reduces permeability, while excessively high values hinder aqueous solubility^56^. SwissADME calculates consensus Log P as the arithmetic mean of five predictive models. For hymenialdisine and spongiacidin D, the consensus Log P values were 0.26 and 0.39, respectively (Fig. 13). These results suggest high water solubility and hydrophilic character, which may reduce passive diffusion through membranes but improve solubility and reduce metabolic accumulation.

#### Cytochrome P450 enzymes

Cytochrome P450 enzymes, primarily found in the liver and intestines, play a crucial role in the oxidative metabolism of the majority of drugs. Inhibiting these enzymes can cause drug–drug interactions, leading to toxicity or reduced efficacy. Among the five major CYP isoforms, hymenialdisine and spongiacidin D were predicted to inhibit only CYP1A2, while showing no inhibition of CYP2C19, CYP2C9, CYP2D6, and CYP3A4 (Table 8).

**Table 8:** Predicted inhibition of CYP450 enzymes by hymenialdisine and spongiacidin D (SwissADME).

Compound	CYP1A2 inhibitor	CYP2C19 inhibitor	CYP2C9 inhibitor	CYP2D6 inhibitor	CYP3A4 inhibitor
Hymenialdisine	Yes	No	No	No	No
Spongiacidin D	Yes	No	No	No	No

#### Toxicity

Toxicity remains a major cause of drug development failure; therefore, early detection is critical. pkCSM was used to predict five toxicity endpoints (Table 9). Both compounds showed a favorable safety profile, being non-mutagenic (AMES negative), non-cardiotoxic (hERG I/II negative), and non-sensitizing to skin. However, a key difference was observed in hepatotoxicity: spongiacidin D was predicted to have potential liver toxicity, while hymenialdisine showed no hepatotoxicity risk. This finding highlights the need for further in vitro and in vivo safety assessments, particularly for spongiacidin D.

**Table 9:** Predicted toxicity endpoints of hymenialdisine and spongiacidin D (pkCSM).

Compound	AMES toxicity	hERGI inhibitor	hERG II inhibitor	Hepatotoxicity	Skin sensitisation
Hymenialdisine	No	No	No	No	No
Spongiacidin D	No	No	No	yes	No

## Discussion

Marine sponges are well-established sources of structurally varied and biologically active secondary metabolites, a considerable number of which have shown substantial pharmaceutical potential, particularly as anticancer agents^37,38^. In the present study, a comparative investigation was undertaken to evaluate the bioactive potential of three Red Sea sponge species—*Stylissa carteri*, *Hemimycale arabica*, and *Negombata magnifica*. This was achieved through an integrative approach combining extraction efficiency assessment, preliminary phytochemical screening, cytotoxicity assays, in vitro wound healing experiments, LC–MS/MS-based chemical profiling, and molecular docking studies. The overarching objective was to identify sponge species and collection sites yielding extracts and compounds with pronounced cytotoxic properties^57^.

The differential extraction yields observed among the sponges can largely be attributed to the variation in polarity and solubility of their metabolic constituents^58^. The n-butanol fractions consistently yielded the highest mass, most notably in *N. magnifica*, suggesting a rich content of polar metabolites such as amino acids, glycosylated compounds, and sulfated secondary products commonly found in marine taxa^59,60^. Substantial yields were also obtained from the ethyl acetate fractions, implying a significant abundance of semi-polar bioactive molecules like alkaloids, phenolics, and terpenoids—classes of compounds frequently reported in the genera *Stylissa*, *Hemimycale*, and *Negombata*^61–63^. Conversely, the n-hexane fractions showed markedly lower yields, aligning with their primary function of extracting lipophilic constituents such as fatty acids and sterols^64^.

These extraction trends were further supported by preliminary phytochemical analyses, which indicated that non-polar fractions were enriched in sterols, terpenoids, and alkaloids, while polar fractions were predominantly composed of flavonoids, saponins, and phenolic compounds. These phytochemicals are well-documented for their antioxidant, anti-inflammatory, and cytotoxic activities, highlighting the therapeutic potential of the butanol fractions, especially those derived from *S. carteri* and *H. arabica*^63,65^. These findings also support previous observations that solvent polarity significantly influences the extraction of specific classes of bioactive compounds, as highlighted by Athmouni et al.^66^, who demonstrated that polar solvents preferentially isolate phenolics and flavonoids, while non-polar solvents enrich terpenoids. This correlation aligns well with our results, where n-butanol and ethyl acetate fractions—representing polar and semi-polar solvents—yielded distinct metabolite profiles and corresponding biological activities.

In vitro cytotoxic assays demonstrated that both sponge species and collection site markedly influenced cytotoxic and anti-migratory activities. Among all tested samples, the total MeOH:DCM extract of *Stylissa carteri* collected from El Gouna exhibited the strongest cytotoxic effect, producing approximately 80% growth inhibition in the MTT assay with an IC_50_ of 37 µg/mL, and significantly reducing HepG2 colony formation to 20.3 ± 2.1% survival. In parallel, this extract effectively suppressed HepG2 cell migration, resulting in only 35.4 ± 2.1% wound closure—an effect comparable to that of the reference anticancer drug doxorubicin. Future studies will include normal hepatocytes or other relevant non-cancerous cell lines to evaluate cytotoxic selectivity and potential therapeutic safety.

LC–MS/MS profiling of the bioactive extract revealed the presence of several brominated pyrrole–imidazole alkaloids, including hymenialdisine, spongiacidin D, hymenidin, and oroidin. Hymenialdisine has been reported to exhibit potent cytotoxicity against hepatocellular carcinoma (HepG2) and other cancer cell lines, acting through cyclin-dependent kinase inhibition and pro-apoptotic pathways^67^. Spongiacidin D has shown selective cytotoxicity against colon and breast cancer cells, partly via inhibition of glycogen synthase kinase-3β (GSK-3β)^68^. Hymenidin and oroidin are known for their moderate cytotoxicity but also for sensitizing cancer cells to apoptosis, as well as exhibiting anti-metastatic effects by interfering with cell adhesion and migration^69^.

The LC–MS/MS results obtained in this study are consistent with previous reports on *Stylissa carteri* and related species, where brominated alkaloids such as hymenialdisine, oroidin, and spongiacidin derivatives have been repeatedly identified as the principal bioactive constituents^70,71^. Likewise, similar metabolites were described in other Red Sea sponges belonging to the families *Dictyonellidae* and *Halichondriidae*, supporting the chemical reliability of the present findings^72^. The detection of these compounds in the current extract therefore corroborates earlier studies and reinforces the notion that *S. carteri* is a rich source of brominated alkaloids with significant cytotoxicity^73^. Although these alkaloids are proposed as potential contributors to the observed cytotoxic effects, direct causal evidence is lacking, and further studies are needed to correlate individual compounds with bioactivity. Future work will involve fractionation, targeted isolation, and spectrum–effect relationship analysis to determine which metabolites are primarily responsible for the cytotoxic activity.

The potent bioactivity of the El Gouna extract is therefore likely attributable to the synergistic action of these alkaloids in combination with phenolic constituents also detected in the crude extract. Phenolics are known to modulate oxidative stress and enhance apoptosis, thereby potentiating the effects of alkaloids^74^. Comparable synergistic effects between brominated alkaloids and phenolic antioxidants have been documented in other Red Sea sponge extracts^70^. The enhanced cytotoxicity observed in the *Stylissa carteri* extract from El Gouna compared to other collection sites is likely driven by environmental influences such as temperature, salinity, nutrient availability, and light exposure, which are known to modulate sponge secondary metabolism. This aligns with findings from *Xestospongia* spp. where the chemical profiles shifted significantly across geographic locales, particularly in brominated fatty acids and sterols^75^; the Mediterranean sponge *Crambe crambe*, which displays season- and region-linked variations in guanidine alkaloid production^76^; and evidence that sponges like *Dysidea avara* exhibit chemically mediated responses to local biotic and abiotic stressors^77^, as well as variations with depth and habitat influencing metabolite distribution^78^.

In silico docking and molecular dynamics studies targeting checkpoint kinase 2 (Chk2) were employed as hypothesis-generating tools to rationalize the observed cytotoxicity and to prioritize the identified alkaloids for further investigation. It is important to emphasize that Chk2 involvement in the observed cytotoxic effects is inferred from computational docking, MD simulations, and MM/GBSA analyses, and therefore represents a mechanistic hypothesis rather than direct experimental validation. While the favorable binding modes, structural stabilization, and energetic profiles strongly suggest Chk2 as a plausible molecular target for hymenialdisine and spongiacidin D, biochemical confirmation of target engagement is required. Accordingly, future studies will focus on in vitro Chk2 kinase inhibition assays and downstream pathway analyses to experimentally validate this proposed mechanism.

MD simulations revealed that ligand binding stabilized the Chk2 structure, as evidenced by reduced RMSD and RMSF values, indicating suppressed backbone fluctuations. Decreases in the radius of gyration (Rg) and solvent-accessible surface area (SASA) further suggested the formation of more compact and structurally stable protein–ligand complexes. Collectively, these parameters support favorable and persistent binding of hymenialdisine and spongiacidin D within the Chk2 catalytic domain. MM/GBSA energy decomposition showed that van der Waals interactions were the dominant contributors to complex stabilization, complemented by electrostatic interactions. Although desolvation penalties partially opposed binding, strong gas-phase interactions resulted in overall favorable binding free energies. Key hydrophobic contacts involving Leu15, Val23, and Leu128, together with stabilizing hydrogen bonds formed by Glu76 and Met78, further reinforced ligand binding. Similar energetic profiles have been reported for kinase–inhibitor systems, where hydrophobic packing within the ATP-binding pocket and complementary hydrogen bonding interactions are recognized as critical determinants of cytotoxic activity, supporting the validity of the present observations.

The ligand–residue interaction map highlighted the synergistic contribution of hydrophobic packing and hydrogen bonding in preserving a favorable binding orientation. Such interactions are frequently recognized as essential determinants of high-affinity and selective kinase inhibition^50,51^. Consistent with this, PCA analyses revealed that ligand binding constrained protein flexibility, thereby limiting conformational fluctuations and ensuring stable occupancy of the active site. Notably, hymenialdisine exhibited stronger binding affinity than spongiacidin D, supported by more persistent hydrogen bonding and favorable hydrophobic contacts, which may explain its higher stabilizing effect within the active site. These findings are in line with previous studies that identified hymenialdisine as a potent kinase inhibitor targeting CDKs, GSK-3β, and PI3K/Akt signaling, with well-documented anticancer potential^67^. By contrast, spongiacidin D, while displaying slightly lower binding affinity, has been reported as a cytotoxic pyrrole–imidazole alkaloid with activity against multiple cancer cell lines^68^. Thus, hymenialdisine can be regarded as a targeted kinase-focused scaffold, whereas spongiacidin D exerts a broader cytotoxic effect, highlighting the chemical diversity of sponge-derived metabolites and their distinct modes of anticancer action. Collectively, these findings suggest that hymenialdisine and spongiacidin D exert their anticancer effects through complementary mechanisms—kinase inhibition and general cytotoxicity, respectively—and their structural stabilization within the Chk2 binding pocket underscores their potential as lead scaffolds for the preclinical development of marine-derived cytotoxic agents.

While these computational results indicate favorable binding modes and stability for hymenialdisine and spongiacidin D, they do not constitute experimental validation of target engagement. Instead, they provide a strong rationale for future experimental work. The logical next step is the bioassay-guided isolation of these lead alkaloids, followed by in vitro kinase inhibition assays against Chk2 to directly test the binding hypothesis generated here. Subsequent mechanistic studies could then explore downstream effects on DNA damage signaling and cell cycle checkpoints in HepG2 cells.

Taken together, the integrative in vitro–in silico approach employed here highlights *S. carteri*—particularly the El Gouna population—as a promising reservoir of cytotoxic agents. The consistency of its bioactivity across MTT, colony formation, and wound healing assays, coupled with supportive chemical and docking evidence, reinforces its pharmaceutical potential. Furthermore, these findings underscore the role of environmental and geographic factors in shaping the secondary metabolite repertoire and, consequently, the bioactive profiles of Red Sea sponges^76^.

## Conclusion

This study identifies *Stylissa carteri* as a promising source of bioactive pyrrole–imidazole alkaloids with notable cytotoxic potential. The El Gouna total MeOH:DCM extract exhibited the strongest cytotoxic, anti-proliferative, and anti-migratory effects against HepG2 cells, correlating with its high phenolic and flavonoid content. LC–MS/MS profiling highlighted hymenialdisine and spongiacidin D, both of which showed favorable binding to Chk2 kinase. Docking, MM/GBSA, and molecular dynamics analyses confirmed van der Waals-driven stabilization, with hymenialdisine displaying stronger affinity and more persistent interactions than spongiacidin D. These findings prioritize *S. carteri* and its constituent alkaloids, particularly hymenialdisine, for further experimental validation, including compound isolation and direct target engagement assays, to advance their development as marine-derived cytotoxic scaffolds.

## Supplementary Information

Below is the link to the electronic supplementary material.

Supplementary Material 1

## Data Availability

All data generated or analysed during this study are included in this published article.
