# Supplementary material for: Cytotoxic activity of marine derived bioactive compounds from red sea sponges supported by LC-MS/MS profiling and molecular docking
Source: Sci Rep. 2026 Mar 12;16:8949. doi: 10.1038/s41598-026-39782-z (PMC12988191; doi:10.1038/s41598-026-39782-z)
Supplement: Supplementary file 1 — Supplementary Material 1 [file 41598_2026_39782_MOESM1_ESM.docx]

**Cytotoxic activity of marine derived bioactive compounds from Red Sea sponges supported by LC-MS/MS profiling and molecular docking**

**Noha E. Ibrahim ^1^, Amal M. El-Feky ^2*^, Mohamed Aboelmagd ^2^, Nadia A. Mohammed ^3^ , Rehab A. Mohamed ^3^, Ahmed A. El‑Rashedy ^4,5^, Hanaa M. Rady ^6^**

^1^ Microbial Biotechnology Department, National Research Centre, 33 El Bohouth St. (Former El Tahrir St.), P.O. 12622, Dokki, Giza, Egypt.

^2^ Pharmacognosy Department, National Research Centre, 33 El Bohouth St. (Former El Tahrir St.), P.O. 12622, Dokki, Giza, Egypt.

^3^ Medical Biochemistry Department, National Research Centre, 33 El Bohouth St. (Former El Tahrir St.), P.O. 12622, Dokki, Giza, Egypt.

^4^ Natural and Microbial Products Department, National Research Center, 33 El Bohouth St. (Former El Tahrir St.), Dokki, P.O. 12622, Giza, Egypt, ^5^ Department Organic and Medicinal Chemistry, Faculty of Pharmacy, University of Sadat City, Menoufia, 32897, Egypt

^6^ Chemistry of Natural Compounds Department, National Research Centre, 33 El Bohouth St. (Former El Tahrir St.), P.O. 12622, Dokki, Giza, Egypt.

Correspondence: Amal M. El-Feky , email: [ammelfeky@hotmail.com](mailto:ammelfeky@hotmail.com)

**
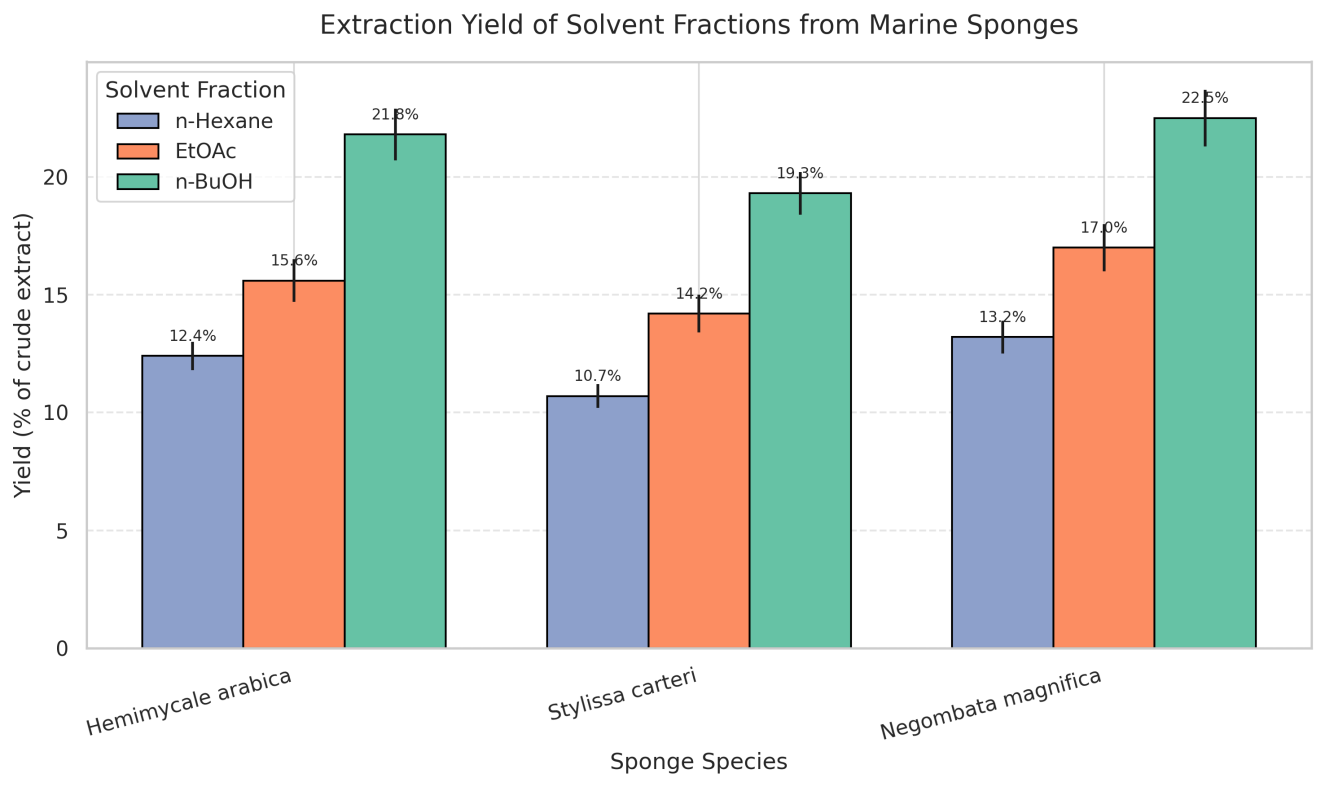
 Figure S1.** Extraction yields (% w/w of crude extract) of sequential solvent fractions (n-hexane, ethyl acetate, and n-butanol) obtained from three marine sponge species: Hemimycale arabica, Stylissa carteri, and Negombata magnifica. Values are expressed as mean ± SD (n = 3). Bars with different **lowercase letters (a–c)** differ significantly among solvent fractions within the same species, while **uppercase letters (A–C)** indicate significant differences for the same solvent among species (p < 0.05, one-way ANOVA followed by Tukey’s post hoc test). Error bars represent standard deviations.

**Table S1:** Summary of docking results using autodock vein for the identified compounds in *Stylissa carteri* total extract with the chk2 receptor.

| **ligands** | ***Hydrogen bonds between atoms of ligands and amino acids of receptor*** | | | | | **S- score**  (binding energy)  (kcal/mol) |
| --- | --- | --- | --- | --- | --- | --- |
|  | ligands  Atoms | Receptor | | Type | Distance (Å) |  |
|  |  | Atoms | Residues |  |  |  |
| Hymenialdisine  (1) | H 2175 | O 992 | GLU351 | H-don | 2.13 | -8.35 |
|  | H 2174 | O 992 | THR 367 | H-don | 1.87 |  |
| Spongiacidin D  (2) | O 4390 | N 1253 | MET 304 | H-acc | 2.05 | -7.30 |
| Dermacozine H (3) | N 27 | OD2 | ASP 311 | H-don | 3.03 | -6.53 |
|  | O 32 | O | Glu302 | H-don | 2.71 |  |
| Hymenidin(4) | N26 | OD1 | Asn 352 | H-don | 3.23 | -6.42 |
|  | N22 | N | Cys231 | H-acceptor | 3.22 |  |
| Oroidin(5) | O9 | N | Met304 | H-acceptor | 3.24 | -6.55 |
|  | 5-ring | CB | Leu226 | Pi-H | 4.08 |  |
| Debromohymenialdisine(6) | 5-ring | CB | Leu226 | Pi-H | 4.00 | -5.86 |
